# Supplementary material for: Changes in the Dentate Gyrus Gene Expression Profile Induced by Levetiracetam Treatment in Rats with Mesial Temporal Lobe Epilepsy
Source: Int J Mol Sci. 2024 Jan 30;25(3):1690. doi: 10.3390/ijms25031690 (PMC10855401; doi:10.3390/ijms25031690)
Supplement: Supplementary file 1 [file ijms-25-01690-s001.zip › ijms-2802386-supplementary.pdf]

S1. TABLE 1. Complete list of DEGs (comparison EPI vs CTRL groups)

ID Gen	Gen	Gene name	FC	P- Value
17786724	A2m	alpha-2-macroglobulin	2.65	0.0003
17796232	Abcc9	ATP-binding cassette, subfamily C (CFTR/MRP), member 9	2.17	9.75E-05
17688180	Abcg3l3	ATP-binding cassette, subfamily G (WHITE), member 3-like 3	-2.18	8.19E-05
17855233	Acpp	acid phosphatase, prostate	-2.16	2.04E-06
17799044	Actl7a	actin-like 7a	-2	2.71E-07
17874255	Actrt1	actin-related protein T1	-2.28	4.89E-06
17772416	Acvr1c	activin A receptor, type IC	2.79	0.0015
17681113	Angptl1	angiopoietin-like 1	-2.01	0.0006
17794610	Apobec1	apolipoprotein B mRNA editing enzyme, catalytic polypeptide 1	2.66	4.20E-07
17853855	Aqp9	aquaporin 9	2.01	0.0011
17795984	Arhgdib	Rho, GDP dissociation inhibitor (GDI) beta	2.35	5.70E-06
17659257	Asic2	acid-sensing (proton-gated) ion channel 2	-2.31	4.20E-05
17684270	Atp2b4	ATPase, Ca++ transporting, plasma membrane 4	-3.89	0.0005
17801412	Best4	bestrophin 4	2.04	6.35E-05
17875011	Bgn	biglycan	3.4	0.0041
17869920	C1ql2	complement component 1, q subcomponent-like 2	-2.83	0.0118
17870022	C3	complement component 3	4.25	2.31E-04
17752965	C4b; C4a; LOC103689965	complement component 4B (Chido blood group); complement component 4A (Rodgers blood group); complement C4-like	2.31	1.07E-06
17660126	Cacna1g	calcium channel, voltage-dependent, T type, alpha 1G subunit	2.27	0.016
17722230	Camk4	calcium/calmodulin-dependent protein kinase IV	-2.27	8.65E-05
17673923	Camkk2	calcium/calmodulin-dependent protein kinase kinase 2, beta	-2.01	0.0021
17784528	Capg	capping protein (actin filament), gelsolin-like	2.18	0.0021
17650231	Car10	carbonic anhydrase 10	2.3	0.0202
17746231	Car13	carbonic anhydrase 13	2.46	3.42E-06
17729278	Cbfb	core-binding factor, beta subunit	2.57	0.0175
17728353	Cbln1	cerebellin 1 precursor	-2.6	0.0038
17711779	Ccdc110	coiled-coil domain containing 110	-2.27	8.02E-09
17798390	Ccin	calicin	-2.15	7.42E-06
17619231	Cckbr	cholecystokinin B receptor	-2.1	0.0065
17794897	Cd4	Cd4 molecule	2.77	0.0003
17632781	Cd37	CD37 molecule	2.48	3.49E-06
17681998	Cd48	Cd48 molecule	2.85	0.0002
17657767	Cd68	Cd68 molecule	2.11	1.47E-07
17723564	Cd74	Cd74 molecule, major histocompatibility complex, class II invariant chain	2.78	3.78E-06
17735497	Cd180	CD180 molecule	2.43	6.90E-08
17681972	Cd244	Cd244 molecule, natural killer cell receptor 2B4	2.06	0.0197
17613244	Cdc42ep5	CDC42 effector protein (Rho GTPase binding) 5	2.02	0.0004
17630680	Ceacam1	carcinoembryonic antigen-related cell adhesion molecule 1 (biliary glycoprotein)	2.69	0.0003
17729229	Ces2h	carboxylesterase 2H	2.39	5.60E-07
17867464	Cesl1	carboxylesterase-like 1	-2.22	1.12E-07
17850971	Chek1	checkpoint kinase 1	2.25	6.23E-05
17782474	Chrm2	cholinergic receptor, muscarinic 2	-2.09	0.0157
17666537	Cldn1	claudin 1	-2.63	0.0004
17786952	Clec4a3	C-type lectin domain family 4, member A3	2.21	3.93E-06
17786978	Clec4b2	C-type lectin domain family 4, member B2	-2.04	4.06E-08

17795399	Clec7a	C-type lectin domain family 7, member A	3.05	0.0001
17843445	Clmp	CXADR-like membrane protein	-2.71	0.0016
17797445	Cngb3	cyclic nucleotide gated channel beta 3	-2.06	6.89E-08
17866373	Col6a3	collagen, type VI, alpha 3	2.38	0.0291
17737360	Cp	ceruloplasmin (ferroxidase)	2.28	0.0005
17723923	Cplx4	complexin 4	4.15	0.0069
17774623	Creb3l1	cAMP responsive element binding protein 3-like 1	2.41	0.0003
17723614	Csf1r	colony stimulating factor 1 receptor	2.11	4.13E-07
17666037	Csta	cystatin A (stefin A)	-3.4	1.19E-05
17859795	Ctla4	cytotoxic T-lymphocyte-associated protein 4	-2.14	1.85E-05
17786463	Cxcl12	chemokine (C-X-C motif) ligand 12	-4.11	0.0083
17614510	Cyp2b3	cytochrome P450, family 2, subfamily b, polypeptide 3	-2.49	0.0008
17614538	Cyp2b12	cytochrome P450, family 2, subfamily b, polypeptide 12	-2.19	1.17E-06
17792700	Cyp26b1	cytochrome P450, family 26, subfamily b, polypeptide 1	-2.83	2.86E-08
17778085	Cyss	cystatin S	-2.27	0.0306
17741754	Dennd2d	DENN/MADD domain containing 2D	2.11	4.12E-06
17775114	Depdc7	DEP domain containing 7	2.05	9.48E-08
17829407	Deptor	DEP domain containing MTOR-interacting protein	-2.96	0.0007
17829410	Deptor	DEP domain containing MTOR-interacting protein	-2.09	0.0052
17714592	Drd1	dopamine receptor D1	-2.01	0.0013
17718588	Ecm2	extracellular matrix protein 2, female organ and adipocyte specific	2.04	0.0004
17877966	Eda2r	ectodysplasin A2 receptor	2.04	1.36E-08
17705094	Ednrb	endothelin receptor type B	2.21	5.08E-05
17866869	Efna5	ephrin A5	2.04	0.0002
17633019	Emp3	epithelial membrane protein 3	2.79	0.0022
17782932	Ephb6	Eph receptor B6	2.3	0.0219
17686529	Fcgr2b	Fc fragment of IgG, low affinity IIb, receptor	2.19	0.0007
17777585	Fermt1	fermitin family member 1	-2.52	1.24E-05
17693089	Fgf5	fibroblast growth factor 5	-2.49	5.10E-07
17680226	Fmod	fibromodulin	2.33	0.0041
17878425	Fnd3c2	fibronectin type III domain containing 3C2	2.12	0.001
17631808	Fxyd7	FXYD domain-containing ion transport regulator 7	-2.91	0.0013
17813224	Gabrd	gamma-aminobutyric acid (GABA) A receptor, delta	-2.37	0.0023
17668380	Gabrr3	gamma-aminobutyric acid (GABA) A receptor, rho 3	-2.29	0.0371
17798651	Galnt12	polypeptide N-acetylgalactosaminyltransferase 12	-2.4	4.12E-06
17814108	Galnt14	polypeptide N-acetylgalactosaminyltransferase 14	-2.34	0.0022
17745158	Gapt	Grb2-binding adaptor protein, transmembrane	2.15	4.05E-05
17641030	Gcnt1	glucosaminyl (N-acetyl) transferase 1, core 2	3.03	0.0009
17706452	Gdf10	growth differentiation factor 10	-2.45	0.0092
17661998	Gfap	glial fibrillary acidic protein	2.25	0.0002
17707706	Glra3	glycine receptor, alpha 3	2.18	4.52E-06
17855604	Gnat1	guanine nucleotide binding protein (G protein), alpha transducing activity polypeptide 1	-2.17	1.18E-05
17879324	Gpc3	glypican 3	-3.2	0.0173
17775480	Grem1	gremlin 1	-2.33	4.27E-07
17750426	Gstm3	glutathione S-transferase mu 3	2.17	0.0006
17750393	Gstm6l	glutathione S-transferase, mu 6-like	2.19	6.10E-06
17785675	Gxylt2	glucoside xylosyltransferase 2	2.52	0.0002
17703201	Gzmbl3; Gzmb	Granzyme B-like 3; granzyme B (granzyme 2, cytotoxic T-lymphocyte-associated serine esterase 1)	-2.59	0.0001

17655129	Hba1	hemoglobin, alpha 1	-4.55	0.0004
17788960	Hgf	hepatocyte growth factor	2.54	0.0029
17715802	Hist1h4b	histone cluster 1, H4b	2.67	2.54E-09
17711526	Hpgd	hydroxyprostaglandin dehydrogenase 15 (NAD)	2.54	7.80E-06
17683699	Htr5b	5-hydroxytryptamine (serotonin) receptor 5B	-2.1	0.0029
17803116	Id3	inhibitor of DNA binding 3	2.02	2.39E-06
17747002	Il2	interleukin 2	-2.1	9.96E-08
17628292	Ipcef1	interaction protein for cytohesin exchange factors 1	-2.04	0.0117
17862411	Kat2b	K(lysine) acetyltransferase 2B	2.09	1.35E-05
17821210	Kcnf1	potassium channel, voltage-gated modifier subfamily F, member 1	-2.31	0.0003
17642469	Kcnip2	Kv channel-interacting protein 2	-2.49	9.44E-05
17639991	Kcnk4	potassium channel, two pore domain subfamily K, member 4	-2.19	0.0027
17873685	Klhl4	kelch-like family member 4	-2.57	0.0075
17725099	Klhl14	kelch-like family member 14	-3.43	7.74E-05
17787545	Klrb1	killer cell lectin-like receptor subfamily B, member 1	2.4	5.51E-06
17666621	Kng1	kininogen 1	-2.2	5.06E-05
17761968	Kynu	kynureninase	-2.31	1.58E-08
17840705	Lalba	lactalbumin, alpha	-2.14	4.65E-08
17766641	Lamp5	lysosomal-associated membrane protein family, member 5	-2.2	0.0332
17673113	Lat2	linker for activation of T cells family, member 2	2.26	3.23E-06
17690315	Ldb2	LIM domain binding 2	-2.25	0.0002
17824104	LOC299277	similar to serine (or cysteine) peptidase inhibitor, clade A, member 3B	2.05	0.0175
17864940	LOC301444	pseudogene for diazepam binding inhibitor 1	-2.41	1.06E-06
17862467	LOC302192; LOC689479; LOC681180	similar to RIKEN cDNA 1700001E04; similar to Discs large homolog 5 (Placenta and prostate DLG) (Discs large protein P-dlg)	-2.15	0.0084
17869395	LOC363301	hypothetical LOC363301	-2.32	0.0001
17854179	LOC367117	similar to RIKEN cDNA 2900055D03	2.02	6.69E-05
17686562	LOC498276; LOC100362543; LOC103693683	Fc gamma receptor II beta; Low affinity immunoglobulin gamma Fc region receptor III-like; low affinity immunoglobulin gamma Fc region receptor III [Source:RGD Symbol;Acc:9127992]; low affinity immunoglobulin gamma Fc region receptor III	2.32	1.10E-06
17697369	LOC498465; LOC102546495	similar to RIKEN cDNA 1700001F09; disks large homolog 5-like	-2.5	0.0005
17738224	LOC499607	similar to GTPase activating protein testicular GAP1	-2.4	1.06E-05
17838174	LOC680316	similar to Cytochrome P450 11B1, mitochondrial precursor (CYPXIB1) (P450C11) (Steroid 11-beta-hydroxylase) (P450(11 beta)-DS)	-2.25	0.0339
17731296	LOC681364	similar to RIKEN cDNA 5031410I06	2.52	9.01E-06
17728554	LOC685411	similar to spermatogenesis associated glutamate (E)-rich protein 4b [Source:RGD Symbol;Acc:1593845]	-2.02	0.0279
17711886	LOC685829; Calcoco2	similar to calcium binding and coiled-coil domain 2; calcium binding and coiled-coil domain 2 [Source:MGI Symbol;Acc:MGI:1343177]	2.58	0.0038
17694635	LOC685935	similar to splicing factor, arginine/serine-rich 10 (transformer 2 homolog, Drosophila)	2.19	0.0034
17631655	LOC688869; Cox6b1	similar to cytochrome c oxidase, subunit VIb polypeptide 1; cytochrome c oxidase subunit VIb polypeptide 1 [Source:RGD Symbol;Acc:1584097]	-2.47	0.0003
17812285	LOC691162	hypothetical protein LOC691162	-2.28	8.50E-07
17656451	LOC691286	similar to RIKEN cDNA 4930504O13	2.25	3.97E-08
17704136	LOC691988; LOC102550654	similar to a disintegrin and metalloproteinase domain 28; disintegrin and metalloproteinase domain-containing protein 28-like	-3.13	6.83E-07
17658276	LOC691995	hypothetical protein LOC691995	2.71	0.0349
17801011	LOC100362122	selection and upkeep of intraepithelial T cells 3-like	2.12	0.0001

17880699	LOC100362391	NADH dehydrogenase (ubiquinone) 1 beta subcomplex 3-like [Source:RGD Symbol;Acc:2318296]	-2.76	7.89E-10
17843065	LOC100362690	rCG64164-like	2.12	6.55E-07
17799414	LOC100362745	zinc finger protein 101-like	-2.14	1.70E-06
17849819	LOC100363016	methyl-CpG binding domain protein 3-like 2-like	-2.07	1.33E-08
17664501	LOC100363287	RIKEN cDNA 2310034C09-like	2.1	0.0002
17874554	LOC100363769; LOC100360296	BRCA2-interacting protein-like; BRCA2-interacting protein-like [Source:RGD Symbol;Acc:2322455]	2.24	1.59E-05
17873615	LOC100363993	testis expressed gene 16-like	2.72	7.75E-06
17822564	LOC100364391; Dhrs7l1	dehydrogenase/reductase (SDR family) member 7-like; dehydrogenase/reductase (SDR family) member 7-like 1 [Source:RGD Symbol;Acc:1308036]	2.26	1.06E-08
17627821	LOC100365542; 1700020N01Rik	rCG41957-like; RIKEN cDNA 1700020N01 gene [Source:MGI Symbol;Acc:MGI:1914942]	-2.23	0.0002
17645248	LOC100365583; LOC100359777	mCG12681-like	2.39	3.03E-05
17877682	LOC100909486	putative SMEK homolog 3-like	2.72	5.75E-07
17857168	LOC100910668; LOC100911489	uncharacterized LOC100910668; uncharacterized LOC100911489	2.15	6.32E-07
17727712	LOC100910721	60S ribosomal protein L26-like	2.08	0.0435
17873629	LOC100911639	uncharacterized LOC100911639; uncharacterized LOC100911639 [Source:RGD Symbol;Acc:6491583]	2.05	0.0004
17740508	LOC100911982	late cornified envelope protein 3C-like	2.67	1.77E-06
17640654	LOC100912336; 1700025F22Rik	membrane-spanning 4-domains subfamily A member 12-like; RIKEN cDNA 1700025F22 gene [Source:MGI Symbol;Acc:MGI:1916666]	-2.25	0.0013
17630502	LOC100912839	CD177 antigen-like	-3.97	4.31E-09
17642957	LOC102546997	uncharacterized LOC102546997	2.22	0.0004
17788342	LOC102547920	zinc finger protein 729-like	-2.18	1.13E-07
17880301	LOC102548248	calphotin-like	2.33	0.0007
17752880	LOC102549726	uncharacterized LOC102549726 [Source:RGD Symbol;Acc:7707260]	-2.02	0.0352
17769413	LOC102550609	zinc finger protein 669-like	-2.48	0.0007
17613800	LOC102550682	carcinoembryonic antigen-related cell adhesion molecule 5-like	-2.23	7.80E-09
17877937	LOC102551035	serine/arginine repetitive matrix protein 4-like	2.21	0.0012
17742664	LOC102553088	collagen alpha-1(XXV) chain-like	-2.41	0.0012
17849254	LOC102553278	zinc finger protein 709-like	2.92	0.0009
17773798	LOC102553814	serine/arginine repetitive matrix protein 1-like; serine/arginine repetitive matrix protein 1-like [Source:RGD Symbol;Acc:7718479]	-2.13	0.0005
17697690	LOC102554774	disks large homolog 5-like	-3.9	5.10E-06
17675117	LOC102554944	CD209 antigen-like protein C-like	-2.03	4.71E-05
17633543	LOC103691126; LOC103691137	uncharacterized LOC103691126; uncharacterized LOC103691137	2.06	0.0037
17807347	LOC103692360; RGD1562165	uncharacterized LOC103692360; similar to GABA(A) receptor-associated protein like 2 [Source:RGD Symbol;Acc:1562165]	2.13	9.44E-06
17769960	LOC103692747	uncharacterized LOC103692747	-2.4	0.0002
17701660	LOC103693372	histone H3.3-like	2.15	6.88E-07
17726248	Lox	lysyl oxidase	4.96	0.0009
17700007	Lpar6	lysophosphatidic acid receptor 6	2	3.52E-05
17777037	Mal	mal, T-cell differentiation protein	-2.16	0.0007
17781647	Mdfic	MyoD family inhibitor domain containing	2.1	0.0007
17645900	Meikin	meiotic kinetochore factor	2.24	4.95E-08
17732777	Mgat4d	MGAT4 family, member D	2.34	1.20E-06
17714181	Mir7a-1	microRNA 7a-1	2.01	0.0002

17860392	Mir26b; Ctdsp1	microRNA 26b; CTD (carboxy-terminal domain, RNA polymerase II, polypeptide A) small phosphatase 1	-2.02	5.27E-07
17740219	Mir190b	microRNA 190b	2.55	8.80E-05
17667243	Mir301b	microRNA 301b	2.08	1.74E-07
17663879	Mir338	microRNA 338	2.24	2.44E-06
17722380	Mir1949	microRNA 1949	-2.81	0.0014
17824340	Mir3579	microRNA 3579	2.36	0.0003
17714890	Mirlet7f-1	microRNA let-7f-1	3.88	0.0026
17738845	Mme	membrane metallo-endopeptidase	-2.3	0.0119
17768956	Mmp9	matrix metallopeptidase 9	2.8	5.00E-04
17633297	Mrgprx2	MAS-related GPR, member X2	2.15	1.20E-03
17624178	Ms4a6a	membrane-spanning 4-domains, subfamily A, member 6A	2.34	0.0019
17640717	Ms4a6b	membrane-spanning 4-domains, subfamily A, member 6B	2.52	1.26E-06
17872868	Msn	moesin	2.11	0.0024
17742517	Ndst4	N-deacetylase/N-sulfotransferase (heparan glucosaminyl) 4	-2.99	1.02E-09
17730510	Necab2	N-terminal EF-hand calcium binding protein 2	-3.03	0.0008
17728443	Neto2	neuropilin (NRP) and tolloid (TLL)-like 2	-2.17	1.24E-05
17686699	Nhlh1	nescient helix loop helix 1	-4.55	0.0058
17634454	Nmb	neuromedin B	-5.8	1.70E-04
17639411	Npas4	neuronal PAS domain protein 4	-2.05	0.0004
17798725	Nr4a3	nuclear receptor subfamily 4, group A, member 3	-2.39	0.0007
17778304	Nrsn2	neurensin 2	-2.35	0.0382
17795055	Ntf3	neurotrophin 3	-2.38	0.0167
17878844	Nxf2	nuclear RNA export factor 2	-2.11	1.18E-07
17718561	Ogn	osteoglycin	2.14	0.0013
17634772	Olr24	olfactory receptor 24	-2.63	1.78E-04
17634774	Olr27	olfactory receptor 27	-2.28	0.0028
17619082	Olr83	olfactory receptor 83	-2.19	0.0002
17635594	Olr126	olfactory receptor 126	2.22	0.0128
17635727	Olr181	olfactory receptor 181	-2.57	2.28E-07
17619335	Olr231	olfactory receptor 231	-2.01	6.19E-06
17635982	Olr251	olfactory receptor 251	2.05	0.0001
17635991	Olr257	olfactory receptor 257	2.45	0.0068
17619415	Olr260	olfactory receptor 260	2.07	0.0141
17638167	Olr295	olfactory receptor 295	-2.04	3.03E-05
17622001	Olr309	olfactory receptor 309	-2.3	0.0001
17624331	Olr346	olfactory receptor 346	2.76	2.24E-06
17624337	Olr352	olfactory receptor 352	2.02	0.0002
17624345	Olr363	olfactory receptor 363	2.02	0.0003
17624351	Olr367	olfactory receptor 367	-2.71	2.09E-07
17624353	Olr371	olfactory receptor 371	2.22	0.0007
17761757	Olr397	olfactory receptor 397	2.21	1.51E-05
17763564	Olr443	olfactory receptor 443	2.42	0.0027
17774112	Olr476	olfactory receptor 476	-2.35	0.0003
17774134	Olr491	olfactory receptor 491	-2.33	1.10E-05
17774158	Olr510	olfactory receptor 510	-2.46	8.97E-05
17763615	Olr515	olfactory receptor 515	2.01	0.0048
17774168	Olr522	olfactory receptor 522	2.52	0.0481
17763658	Olr566	olfactory receptor 566	2.36	6.61E-05
17774222	Olr576	olfactory receptor 576	-2.11	0.0022

17774226	Olr578	olfactory receptor 578	2.71	0.0137
17774228	Olr584	olfactory receptor 584	2.24	0.0044
17763680	Olr586	olfactory receptor 586	-2.2	4.77E-05
17763689	Olr594	olfactory receptor 594	2.21	3.82E-05
17774242	Olr609	olfactory receptor 609	-2.1	1.71E-05
17774357	Olr711	olfactory receptor 711	2	0.0329
17764606	Olr749	olfactory receptor 749	-2.57	1.42E-07
17775327	Olr790	olfactory receptor 790	2.96	2.00E-04
17807273	Olr847; LOC100912327	olfactory receptor 847; olfactory receptor 13C3-like	-2.68	2.42E-07
17801552	Olr855	olfactory receptor 855	-2.1	0.0235
17850014	Olr1149	olfactory receptor 1149	2.34	7.41E-05
17842245	Olr1160	olfactory receptor 1160	-2.12	9.86E-05
17842269	Olr1179	olfactory receptor 1179	2.28	0.0005
17843256	Olr1219	olfactory receptor 1219	2.24	0.0067
17843280	Olr1232	olfactory receptor 1232	2	0.025
17843282	Olr1233	olfactory receptor 1233	3.32	8.62E-06
17843304	Olr1244	olfactory receptor 1244	-2.1	1.44E-05
17843320	Olr1251	olfactory receptor 1251	-2.08	6.00E-03
17843357	Olr1262	olfactory receptor 1262	2.39	0.0002
17851236	Olr1273	olfactory receptor 1273	2.6	9.86E-05
17851260	Olr1292	olfactory receptor 1292	-2.11	0.0001
17851268	Olr1297	olfactory receptor 1297	2.09	0.0047
17843379	Olr1308	olfactory receptor 1308	2.8	1.78E-05
17851371	Olr1338	olfactory receptor 1338	2.1	1.40E-05
17654078	Olr1378	olfactory receptor 1378	2.03	0.0029
17656475	Olr1423	olfactory receptor 1423	-2.09	1.96E-06
17646233	Olr1456	olfactory receptor 1456	-2.54	0.0006
17648343	Olr1493	olfactory receptor gene Olr1493	2.05	0.0001
17648363	Olr1505	olfactory receptor 1505	-2.18	0.008
17648368	Olr1511	olfactory receptor 1511	4.08	0.0002
17648378	Olr1519	olfactory receptor 1519	2.13	0.0007
17665114	Olr1532	olfactory receptor 1532	2.8	0.01
17665142	Olr1559	olfactory receptor 1559	-2.45	3.11E-06
17666901	Olr1566	olfactory receptor 1566	2.01	0.0139
17666909	Olr1570	olfactory receptor 1570	-2.15	3.80E-08
17682192	Olr1583	olfactory receptor 1583	-2.13	6.66E-06
17755904	Olr1671	olfactory receptor 1671	2.29	0.0005
17755924	Olr1686; LOC100910479	olfactory receptor gene Olr1686; olfactory receptor 2G3-like; olfactory receptor 2G3-like [Source:RGD Symbol;Acc:6498308]	-2.33	8.00E-04
17755930	Olr1691	olfactory receptor 1691	-4.3	8.99E-05
17755978	Olr1733	olfactory receptor 1733	2.78	1.10E-06
17747605	P2ry12	purinergic receptor P2Y, G-protein coupled, 12	2.07	1.85E-05
17747603	P2ry13	purinergic receptor P2Y, G-protein coupled, 13	2.04	0.0023
17777209	Pdyn	prodynorphin	-2.68	0.0054
17877202	Phka2	phosphorylase kinase, alpha 2	2.22	4.30E-05
17638696	Phlda2	pleckstrin homology-like domain, family A, member 2	-2.61	0.0001
17726662	Piezo2	piezo-type mechanosensitive ion channel component 2	-2.25	0.0084
17755203	Pkib	protein kinase (cAMP-dependent, catalytic) inhibitor beta	-3.19	0.0002
17833693	Plk5	polo-like kinase 5	-4.81	0.0022

17846617	Plod2	procollagen lysine, 2-oxoglutarate 5-dioxygenase 2	2.39	0.0002
17660675	Plxdc1	plexin domain containing 1	-2.06	0.0273
17873593	Pou3f4	POU class 3 homeobox 4	2.2	4.52E-05
17719311	Prl3d2	Prolactin family 3, subfamily d, member 2	-2.28	0.0015
17790928	Prss58	protease, serine, 58	-2.02	0.0006
17680795	Ptgs2	prostaglandin-endoperoxide synthase 2	2.18	0.0039
17838988	Pvalb	parvalbumin	-2.16	4.71E-07
17652539	Rab37	RAB37, member RAS oncogene family	-2.8	2.90E-05
17814787	Rad51ap2	RAD51 associated protein 2	-2.45	7.94E-09
17691449	Ramp3	receptor (G protein-coupled) activity modifying protein 3	-2.53	0.0013
17728101	Rasd2	RASD family, member 2	-4.06	2.63E-05
17881072	Rbm31y	RNA binding motif 31, Y-linked	-2.01	2.91E-08
17832079	RGD1305928	hypothetical LOC300207	-2.11	1.87E-06
17723515	RGD1309362	similar to interferon-inducible GTPase	3.41	0.0005
17641180	RGD1359158	similar to RIKEN cDNA 1110059E24	-2.22	0.0357
17856971	RGD1559808	similar to 40S ribosomal protein S26	-2.12	4.93E-06
17672704	RGD1560262	similar to cell surface receptor FDFACT	2.78	1.47E-06
17851355	RGD1560789	similar to ribosomal protein S2 [Source:RGD Symbol;Acc:1560789]	2.62	3.17E-06
17808105	RGD1561195	similar to ribosomal protein L31 [Source:RGD Symbol;Acc:1561195]	2.28	4.20E-07
17766256	RGD1561317	similar to ribosomal protein L31 [Source:RGD Symbol;Acc:1561317]	-2.11	3.95E-05
17870973	RGD1561661	similar to Ferritin light chain (Ferritin L subunit)	2.11	8.01E-07
17836696	RGD1561812	similar to Retinol dehydrogenase type II (RODH II) (29 k-protein)	2.2	4.95E-09
17752465	RGD1562652	similar to class I histocompatibility antigen alpha chain - cotton-top tamarin	-2.57	3.23E-09
17852398	RGD1562811	RGD1562811	2.52	0.0012
17872772	RGD1563554	similar to MGC79482 protein	2.08	0.016
17747131	RGD1563562	similar to GTPase activating protein testicular GAP1	-2.02	1.25E-05
17723224	RGD1564428	similar to FLJ32921 protein	-2.26	2.88E-07
17880955	RGD1564447	similar to Zgc:56193	-2.42	4.20E-05
17684828	RGD1564614	similar to complement factor H-related protein	2.01	0.0003
17838872	RGD1565356	similar to RIKEN cDNA 2210421G13	-2.05	2.11E-06
17870530	Rhox9	reproductive homeobox 9	-2.04	4.00E-04
17718745	Rnf182	ring finger protein 182	-2.67	0.0001
17862773	Rrp36	ribosomal RNA processing 36	-2.27	6.75E-05
17668012	Runx1	runt-related transcription factor 1	2.61	1.36E-08
17740581	S100a11	S100 calcium binding protein A11	4.64	0.0271
17642329	Scd1	stearoyl-Coenzyme A desaturase 1	3.13	0.0004
17843753	Scn4b	sodium channel, voltage-gated, type IV, beta subunit	-2.74	6.07E-05
17789123	Sema3d	sema domain, immunoglobulin domain (Ig), short basic domain, secreted, (semaphorin) 3D	2.49	0.0081
17715387	Serpinb1a	serine (or cysteine) proteinase inhibitor, clade B, member 1a	3.44	2.00E-04
17774023	Serping1	serpin peptidase inhibitor, clade G (C1 inhibitor), member 1	2.67	0.0027
17860763	Sgpp2	sphingosine-1-phosphate phosphatase 2	-2.42	0.0055
17807027	Shb	Src homology 2 domain containing adaptor protein B	2.63	0.0489
17777302	Slc4a11	solute carrier family 4, sodium borate transporter, member 11	-4.61	0.0045
17856878	Slc5a7	solute carrier family 5 (sodium/choline cotransporter), member 7	-4.23	0.0198
17842148	Smco4	single-pass membrane protein with coiled-coil domains 4	-2.18	7.32E-05
17872759	Smek3	SMEK homolog 3, suppressor of mek1 (Dictyostelium)	-2.49	0.0094
17836857	Snx31	sorting nexin 31	2.3	0.0002
17656091	Sowaha	sosondowah ankyrin repeat domain family member A	-2.54	0.0011

17821305	Sox11	SRY (sex determining region Y)-box 11	2.87	0.0068
17670964	Spidr	scaffolding protein involved in DNA repair	2.01	7.69E-06
17816003	Sstr1	somatostatin receptor 1	-2.06	0.0048
17805306	Sulf1	sulfatase 1	2.18	0.0014
17807544	Svep1	sushi, von Willebrand factor type A, EGF and pentraxin domain containing 1	-2.01	0.0011
17627744	Taar7d	trace-amine-associated receptor 7d	2.66	5.12E-05
17828745	Tac3	tachykinin 3	12.49	0.0222
17842132	Taf1d	TATA box binding protein (Tbp)-associated factor, RNA polymerase I, D	-2.17	0.0011
17823874	Tc2n	tandem C2 domains, nuclear	-2.52	0.0038
17637963	Tcerg1l	transcription elongation regulator 1-like	-2.68	0.0065
17793044	Tcp1-ps1; Tcp1	t-complex protein 1, pseudogene 1; t-complex 1	-3.76	1.14E-05
17678383	Tesc	tescalcin	2	0.0295
17712159	Tex15	testis expressed 15	-2.51	4.36E-06
17653382	Tex19.1	testis expressed 19.1	2.07	0.0007
17853201	Thsd4	thrombospondin, type I, domain containing 4	-2.13	2.48E-07
17873489	Tlr13	toll-like receptor 13	2.66	7.88E-06
17856033	Tmie	transmembrane inner ear	2.03	2.31E-06
17793337	Trh	thyrotropin releasing hormone	10.67	0.0500
17729538	Tsnaxip1	translin-associated factor X interacting protein 1	2.01	1.58E-05
17774707	Tspan18	tetraspanin 18	-2.36	0.0007
17650493	Ttll6	tubulin tyrosine ligase-like family, member 6	-2.02	2.64E-06
17666520	Uts2b	urotensin 2B	-2.17	7.66E-05
17730371	Vat1l	vesicle amine transport 1-like	2.35	0.0397
17693686	Vcsa1	variable coding sequence A1	-2.12	2.55E-05
17747805	Veph1	ventricular zone expressed PH domain-containing 1	-2.01	6.00E-05
17717148	Vim	vimentin	2.7	0.0129
17629816	Vom1r47	vomeronasal 1 receptor 47	2.11	1.22E-05
17613332	Vom1r53	vomeronasal 1 receptor 53	-2.18	0.0002
17783879	Vom1r66	vomeronasal 1 receptor 66	2.19	3.71E-06
17612647	Vom2r24	vomeronasal 2 receptor, 24	-2.5	6.64E-06
17692353	Vom2r69	vomeronasal 2 receptor, 69	-2.14	0.0003
17612847	Vom2r-ps45	vomeronasal 2 receptor, pseudogene 45	-2.05	0.0007
17692381	Vom2r-ps125	vomeronasal 2 receptor, pseudogene 125	2.32	0.0029
17768299	Vstm2l	V-set and transmembrane domain containing 2 like	-3.72	0.0001
17783679	Wipf3	WAS/WASL interacting protein family, member 3	2.02	0.0138
17823405	Zdhhc22	zinc finger, DHHC-type containing 22	-2.05	2.71E-05
17612499	Zfp53	zinc finger protein 53	2.17	9.00E-03
17773787	Zfp385b	zinc finger protein 385B	-2.07	2.27E-05
17665377	Zpld1	zona pellucida-like domain containing 1	2.64	4.61E-08
17881379		Sequence without official gene name	3.18	1.98E-06
17881044		Sequence without official gene name	-3.9	6.97E-06
17823051		Sequence without official gene name	3.67	1.82E-09
17819373		Sequence without official gene name	-2.06	5.66E-07
17819748		Sequence without official gene name	-2.28	0.0242
17820577		Sequence without official gene name	2.36	0.0005
17881529		Sequence without official gene name	2.12	0.0003
17849245		Sequence without official gene name	-2.48	3.31E-05
17881771		Sequence without official gene name	2.58	8.82E-05

17806985	Sequence without official gene name	-2.25	0.0013
17874764	Sequence without official gene name	3	1.24E-06
17883183	Sequence without official gene name	-2.28	2.23E-08
17874438	Sequence without official gene name	2.35	2.66E-08
17808101	Sequence without official gene name	2.09	0.0047
17874414	Sequence without official gene name	-2.98	4.03E-06
17883025	Sequence without official gene name	2.27	0.0025
17882841	Sequence without official gene name	-2.25	0.0013
17882815	Sequence without official gene name	2.09	0.0003
17808168	Sequence without official gene name	-2.32	0.001
17882813	Sequence without official gene name	-2.15	1.30E-06
17808237	Sequence without official gene name	-2.1	0.0165
17873741	Sequence without official gene name	2.1	0.0004
17882811	Sequence without official gene name	2.16	0.0027
17882441	Sequence without official gene name	2.06	0.0329
17873645	Sequence without official gene name	-2.88	1.52E-05
17876834	Sequence without official gene name	2.74	2.10E-08
17815526	Sequence without official gene name	-2.13	0.0238
17882379	Sequence without official gene name	-3.01	0.0199
17872065	Sequence without official gene name	-2.3	3.83E-06
17816877	Sequence without official gene name	-2.01	0.0012
17817028	Sequence without official gene name	2.08	0.0017
17817688	Sequence without official gene name	3.35	0.0144
17817742	Sequence without official gene name	2.24	0.0003
17817763	Sequence without official gene name	-2.02	0.0201
17818260	Sequence without official gene name	2.15	6.84E-05
17818497	Sequence without official gene name	-2.26	1.72E-07
17818641	Sequence without official gene name	-2.02	0.0016
17818965	Sequence without official gene name	2.17	0.0022
17881711	Sequence without official gene name	-2.4	2.13E-07
17871507	Sequence without official gene name	2.7	0.0167
17867338	Sequence without official gene name	-2.06	0.0019
17824907	Sequence without official gene name	-2.05	0.0004
17857960	Sequence without official gene name	-4.96	0.0079
17858005	Sequence without official gene name	-2.66	1.54E-05
17858107	Sequence without official gene name	-2.23	1.80E-06
17878575	Sequence without official gene name	-2.21	2.46E-05
17859862	Sequence without official gene name	-2.29	5.86E-06
17860134	Sequence without official gene name	-2.41	2.39E-07
17880675	Sequence without official gene name	-2.44	1.57E-06
17843113	Sequence without official gene name	-2.26	0.0212
17862389	Sequence without official gene name	-2	2.90E-06
17862722	Sequence without official gene name	2.08	1.03E-07
17880849	Sequence without official gene name	-2.27	2.90E-07
17863071	Sequence without official gene name	2.22	0.0012
17842140	Sequence without official gene name	-2.22	1.80E-06
17838859	Sequence without official gene name	2.64	2.81E-06
17861007	Sequence without official gene name	2.04	0.0102
17863211	Sequence without official gene name	2.59	7.15E-07
17878675	Sequence without official gene name	3.28	5.85E-07

17880523	Sequence without official gene name	2.32	0.0052
17846338	Sequence without official gene name	2.24	6.00E-08
17880420	Sequence without official gene name	-2.19	8.57E-06
17880438	Sequence without official gene name	2.33	0.0001
17880197	Sequence without official gene name	2.22	0.0003
17846043	Sequence without official gene name	-2.11	0.0005
17844992	Sequence without official gene name	-2.28	0.005
17855026	Sequence without official gene name	2.12	7.27E-08
17844988	Sequence without official gene name	2.59	3.45E-05
17844687	Sequence without official gene name	-2.78	0.0007
17880157	Sequence without official gene name	4.54	8.35E-09
17843984	Sequence without official gene name	3.32	4.28E-05
17880499	Sequence without official gene name	2.25	0.0002
17843745	Sequence without official gene name	2.02	0.0249
17854521	Sequence without official gene name	2.18	0.001
17844818	Sequence without official gene name	-2.37	0.0229
17880952	Sequence without official gene name	-2.24	0.0253
17835409	Sequence without official gene name	2.14	0.0452
17835135	Sequence without official gene name	2.06	0.0364
17825132	Sequence without official gene name	2.07	0.0002
17868881	Sequence without official gene name	2.08	8.18E-05
17877416	Sequence without official gene name	-2.16	0.0185
17868992	Sequence without official gene name	2.31	2.49E-07
17869545	Sequence without official gene name	2.32	1.29E-05
17869572	Sequence without official gene name	-2.68	7.24E-10
17877500	Sequence without official gene name	-2.34	0.023
17869704	Sequence without official gene name	2.53	0.0017
17870356	Sequence without official gene name	-2.44	0.0006
17870607	Sequence without official gene name	-4.43	8.68E-05
17871383	Sequence without official gene name	-2.5	6.31E-06
17871394	Sequence without official gene name	2.18	0.0013
17825125	Sequence without official gene name	-2.71	0.0093
17824953	Sequence without official gene name	-2.17	1.64E-07
17870199	Sequence without official gene name	-2.15	0.012
17825708	Sequence without official gene name	-2.67	0.0004
17827056	Sequence without official gene name	2.03	0.0104
17827534	Sequence without official gene name	2.67	0.0002
17835123	Sequence without official gene name	2.75	0.0065
17834074	Sequence without official gene name	2.06	0.0189
17833256	Sequence without official gene name	-3.42	0.0013
17833233	Sequence without official gene name	-5.75	4.34E-06
17832215	Sequence without official gene name	2.3	0.0007
17865119	Sequence without official gene name	2.46	7.80E-06
17831711	Sequence without official gene name	-2.31	0.0002
17867227	Sequence without official gene name	2.13	0.0244
17867286	Sequence without official gene name	-3.77	4.34E-07
17867320	Sequence without official gene name	-2.05	0.0021
17867324	Sequence without official gene name	2.26	1.64E-07
17867330	Sequence without official gene name	-2.12	0.0114
17867332	Sequence without official gene name	2.12	2.38E-05

17829201	Sequence without official gene name	-2.42	2.25E-05
17867336	Sequence without official gene name	2.12	1.05E-07
17824770	Sequence without official gene name	2.07	0.0008
17875897	Sequence without official gene name	2.31	0.0002
17610316	Sequence without official gene name	-2.98	1.81E-05
17801861	Sequence without official gene name	2.08	6.64E-05
17684940	Sequence without official gene name	-2.18	1.11E-06
17684462	Sequence without official gene name	-3.57	0.0001
17682015	Sequence without official gene name	2.02	9.91E-08
17680711	Sequence without official gene name	-2.08	2.01E-05
17680512	Sequence without official gene name	2.03	0.0035
17677104	Sequence without official gene name	-5.91	3.70E-05
17677101	Sequence without official gene name	-2.03	4.33E-07
17676432	Sequence without official gene name	-2.33	3.35E-07
17674977	Sequence without official gene name	-3.47	3.45E-07
17674965	Sequence without official gene name	-3.58	1.03E-08
17686822	Sequence without official gene name	2.19	0.0006
17674940	Sequence without official gene name	-2.11	7.25E-05
17674936	Sequence without official gene name	-2.07	4.89E-08
17674934	Sequence without official gene name	2.74	0.0027
17674932	Sequence without official gene name	2.12	2.22E-07
17674327	Sequence without official gene name	2.16	0.0013
17673659	Sequence without official gene name	-2.24	0.0025
17673387	Sequence without official gene name	2.26	8.37E-05
17673385	Sequence without official gene name	2.51	0.0042
17671394	Sequence without official gene name	-2.04	0.0002
17671392	Sequence without official gene name	-2.06	5.50E-07
17671390	Sequence without official gene name	2.15	5.55E-05
17674938	Sequence without official gene name	-2.04	0.0032
17687755	Sequence without official gene name	-2.06	0.0003
17688026	Sequence without official gene name	2.27	5.64E-06
17688843	Sequence without official gene name	-2.37	0.0013
17700646	Sequence without official gene name	2.02	1.53E-05
17700462	Sequence without official gene name	2.15	0.0013
17700156	Sequence without official gene name	-2.78	0.0002
17699104	Sequence without official gene name	2.53	2.01E-05
17698372	Sequence without official gene name	2.11	0.0427
17698284	Sequence without official gene name	2.05	0.0024
17698215	Sequence without official gene name	2.57	7.48E-09
17697343	Sequence without official gene name	-2.73	0.0471
17697276	Sequence without official gene name	2.13	0.0004
17697236	Sequence without official gene name	-2.16	1.10E-05
17696972	Sequence without official gene name	2.14	0.001
17696200	Sequence without official gene name	-2.02	3.05E-06
17694916	Sequence without official gene name	-2.01	0.0003
17694807	Sequence without official gene name	-2.02	0.001
17694680	Sequence without official gene name	-2.13	0.0036
17694658	Sequence without official gene name	-2.65	0.0003
17694406	Sequence without official gene name	-3.91	0.0332
17692717	Sequence without official gene name	2.02	0.0154

17690989	Sequence without official gene name	2.08	0.0029
17690412	Sequence without official gene name	-2.16	3.17E-06
17690410	Sequence without official gene name	-3.07	1.61E-05
17689967	Sequence without official gene name	2.2	1.44E-05
17689949	Sequence without official gene name	-2.95	1.08E-07
17670531	Sequence without official gene name	-2.05	2.49E-06
17669287	Sequence without official gene name	2.12	0.0072
17669060	Sequence without official gene name	2.31	1.23E-05
17665061	Sequence without official gene name	2.22	0.0184
17633427	Sequence without official gene name	-2.69	0.0008
17632209	Sequence without official gene name	2.8	3.07E-10
17631881	Sequence without official gene name	-2.34	0.0001
17629813	Sequence without official gene name	-2.42	0.0001
17629742	Sequence without official gene name	2.11	1.95E-06
17629081	Sequence without official gene name	-3.43	3.15E-06
17628801	Sequence without official gene name	-2.2	1.79E-06
17626708	Sequence without official gene name	-2.04	1.88E-07
17626408	Sequence without official gene name	-2.39	2.51E-05
17621278	Sequence without official gene name	-2.76	7.89E-10
17619921	Sequence without official gene name	2.1	0.0008
17619842	Sequence without official gene name	-2.13	1.56E-06
17619502	Sequence without official gene name	2.38	0.0004
17619116	Sequence without official gene name	-2.34	0.0035
17618570	Sequence without official gene name	2.12	0.0045
17615647	Sequence without official gene name	-2.43	5.56E-09
17612377	Sequence without official gene name	2.24	0.0041
17612256	Sequence without official gene name	2.16	0.0408
17612148	Sequence without official gene name	-2.68	0.0003
17611532	Sequence without official gene name	-2.36	1.57E-07
17611073	Sequence without official gene name	-2.13	3.43E-07
17610773	Sequence without official gene name	2.02	0.0009
17610569	Sequence without official gene name	2.04	0.0004
17633531	Sequence without official gene name	-2.14	0.0003
17701035	Sequence without official gene name	2.15	2.21E-08
17633595	Sequence without official gene name	2.19	0.0077
17635699	Sequence without official gene name	-2.63	0.0014
17665017	Sequence without official gene name	-2.19	0.0002
17660267	Sequence without official gene name	-3.06	0.0011
17659966	Sequence without official gene name	-2.2	8.37E-05
17657789	Sequence without official gene name	-2.36	3.13E-06
17657112	Sequence without official gene name	-2.53	0.0037
17655938	Sequence without official gene name	2.17	0.0061
17655103	Sequence without official gene name	-2.8	8.61E-09
17654341	Sequence without official gene name	-2.36	0.0126
17652949	Sequence without official gene name	2.77	3.94E-06
17651986	Sequence without official gene name	2.07	8.29E-05
17650851	Sequence without official gene name	2.64	3.21E-05
17648851	Sequence without official gene name	-2.36	7.09E-06
17647241	Sequence without official gene name	2.25	0.0017
17646015	Sequence without official gene name	-2.28	0.0009

17641707	Sequence without official gene name	-2.46	0.0287
17641187	Sequence without official gene name	2.38	1.94E-07
17640942	Sequence without official gene name	-4.23	6.48E-07
17640876	Sequence without official gene name	2.01	0.0017
17640315	Sequence without official gene name	2.04	8.67E-05
17640313	Sequence without official gene name	-2.01	0.001
17637628	Sequence without official gene name	2.07	2.18E-06
17636409	Sequence without official gene name	-2.38	0.0002
17636356	Sequence without official gene name	2.13	0.0007
17633643	Sequence without official gene name	-2.92	0.0288
17701567	Sequence without official gene name	2.6	0.0027
17702253	Sequence without official gene name	-2.92	0.0288
17702284	Sequence without official gene name	-2.19	7.89E-09
17784236	Sequence without official gene name	-2.97	1.34E-10
17784212	Sequence without official gene name	3.26	1.63E-09
17784165	Sequence without official gene name	-2.83	1.55E-08
17783943	Sequence without official gene name	-2.55	5.70E-05
17783064	Sequence without official gene name	2.08	1.38E-06
17782563	Sequence without official gene name	2.4	4.21E-07
17779667	Sequence without official gene name	2.6	0.0002
17777892	Sequence without official gene name	-2.53	0.0002
17777749	Sequence without official gene name	-2.09	5.10E-07
17777650	Sequence without official gene name	-3.36	0.0191
17777537	Sequence without official gene name	-3.1	6.01E-06
17773351	Sequence without official gene name	2.27	0.0375
17771235	Sequence without official gene name	-2.92	0.0288
17770715	Sequence without official gene name	-2.09	3.35E-05
17769015	Sequence without official gene name	2.46	4.87E-05
17768666	Sequence without official gene name	-2.59	0.032
17766508	Sequence without official gene name	-2.03	4.93E-06
17764642	Sequence without official gene name	2.07	0.0094
17764482	Sequence without official gene name	-2.06	4.89E-05
17762922	Sequence without official gene name	2.74	0.0023
17762116	Sequence without official gene name	2.37	0.0047
17759143	Sequence without official gene name	2.34	0.0011
17756113	Sequence without official gene name	-2.02	6.43E-08
17784275	Sequence without official gene name	-2.17	0.0011
17883199	Sequence without official gene name	2.06	0.0006
17784299	Sequence without official gene name	2.35	0.0058
17785896	Sequence without official gene name	-2.92	0.0288
17801750	Sequence without official gene name	-2.33	0.0088
17799919	Sequence without official gene name	-2.33	0.0088
17799873	Sequence without official gene name	-2.33	0.0088
17799807	Sequence without official gene name	3.35	5.12E-07
17799767	Sequence without official gene name	2.11	0.0002
17799360	Sequence without official gene name	2.13	4.85E-07
17797986	Sequence without official gene name	2.04	0.0126
17797244	Sequence without official gene name	-2.33	0.0088
17796677	Sequence without official gene name	-2.92	0.0288
17796556	Sequence without official gene name	-2.92	0.0288

17796076	Sequence without official gene name	-2.33	0.0088
17793627	Sequence without official gene name	-2.95	6.52E-07
17792793	Sequence without official gene name	-2.36	0.0005
17792454	Sequence without official gene name	-2.7	0.0477
17792022	Sequence without official gene name	-2.59	5.46E-05
17792015	Sequence without official gene name	-2.04	0.0093
17791978	Sequence without official gene name	-2.78	0.0045
17791880	Sequence without official gene name	-2.23	0.0169
17791875	Sequence without official gene name	-3.3	0.0002
17791793	Sequence without official gene name	2.5	7.66E-05
17791614	Sequence without official gene name	-3.11	3.89E-05
17789144	Sequence without official gene name	-2.26	6.86E-06
17789082	Sequence without official gene name	-2.31	0.0004
17784573	Sequence without official gene name	2.46	0.0008
17802894	Sequence without official gene name	-2.25	0.0002
17755310	Sequence without official gene name	-3.73	6.54E-05
17755177	Sequence without official gene name	-2.72	2.59E-06
17727248	Sequence without official gene name	-2.26	0.0027
17726961	Sequence without official gene name	-2.03	0.0019
17726392	Sequence without official gene name	-2.17	6.07E-06
17725929	Sequence without official gene name	2.12	0.0006
17725568	Sequence without official gene name	2.39	0.0334
17723002	Sequence without official gene name	2.18	0.0002
17721679	Sequence without official gene name	-3.53	0.0361
17721677	Sequence without official gene name	-2.53	0.0002
17720724	Sequence without official gene name	2.09	0.0052
17719967	Sequence without official gene name	2.08	2.83E-06
17719767	Sequence without official gene name	3.14	0.0247
17719465	Sequence without official gene name	2.03	1.19E-06
17717138	Sequence without official gene name	-2.61	1.26E-08
17713688	Sequence without official gene name	3.3	2.11E-05
17710544	Sequence without official gene name	2.23	0.0002
17709864	Sequence without official gene name	-2.63	6.19E-07
17708174	Sequence without official gene name	-3.43	5.79E-09
17706987	Sequence without official gene name	2.56	0.0425
17706237	Sequence without official gene name	-2.09	1.03E-08
17704987	Sequence without official gene name	2.02	0.0365
17704845	Sequence without official gene name	-2.09	3.55E-05
17703425	Sequence without official gene name	-2.76	0.0104
17702313	Sequence without official gene name	-2.76	0.0104
17727949	Sequence without official gene name	2.32	0.0102
17755248	Sequence without official gene name	2.06	0.0004
17728515	Sequence without official gene name	2.01	0.0005
17729198	Sequence without official gene name	2.27	0.0001
17755135	Sequence without official gene name	-2.37	0.0002
17755090	Sequence without official gene name	-2.09	0.0147
17752878	Sequence without official gene name	-2.25	9.15E-05
17751015	Sequence without official gene name	2.17	7.35E-05
17750260	Sequence without official gene name	2.84	2.43E-06
17750061	Sequence without official gene name	2.02	5.19E-06

17749162	Sequence without official gene name	-2.08	2.71E-08
17747984	Sequence without official gene name	-2.2	1.38E-05
17747849	Sequence without official gene name	2.13	0.0002
17747675	Sequence without official gene name	-2.47	0.0439
17747033	Sequence without official gene name	-3.09	4.01E-08
17744703	Sequence without official gene name	-2.25	0.0035
17744428	Sequence without official gene name	2.12	0.0162
17744225	Sequence without official gene name	2.06	0.0411
17743763	Sequence without official gene name	-2.26	0.0005
17742063	Sequence without official gene name	2.04	3.78E-05
17739202	Sequence without official gene name	2.18	0.001
17738332	Sequence without official gene name	-2.11	9.68E-05
17736833	Sequence without official gene name	2.65	0.0017
17736667	Sequence without official gene name	2.03	3.36E-07
17736646	Sequence without official gene name	2.32	0.0001
17736023	Sequence without official gene name	-2.03	0.0089
17732173	Sequence without official gene name	2.18	0.0001
17728574	Sequence without official gene name	3.52	0.0016
17883209	Sequence without official gene name	-3.69	0.0007

S2. Table 2. Complete list of DEGs (comparison EPI + LEV vs EPI groups)

ID Gen	Gen	Gen name	FC	P-Value
17696684	A830031A19Rik	RIKEN cDNA A830031A19 gene [Source:MGI Symbol;Acc:MGI:2685508]	-2.13	3.36E-07
17843122	Acrv1	acrosomal vesicle protein 1	-2.09	2.21E-06
17638084	Adam8	ADAM metallopeptidase domain 8	-2.11	3.33E-05
17782314	Akr1b8	aldo-keto reductase family 1, member B8	2.17	0.0345
17795984	Arhgdib	Rho, GDP dissociation inhibitor (GDI) beta	-2.11	3.33E-05
17844083	Btg4	B-cell translocation gene 4	-2.03	1.12E-08
17753110	Btn3a2	butyrophilin, subfamily 3, member A2	-2.53	2.01E-05
17744705	Cartpt	CART prepropeptide	-2.15	0.0137
17744890	Ccnb1	cyclin B1	-2.03	8.67E-05
17694921	Cd38	CD38 molecule	-2.03	0.0001
17621950	Cd163l1	CD163 molecule-like 1	-2.09	8.03E-08
17735497	Cd180	CD180 molecule	-2.03	3.91E-07
17614274	Ceacam10	carcinoembryonic antigen-related cell adhesion molecule 10	-2.12	0.0039
17857860	Cenpq	centromere protein Q	-2.2	1.68E-06
17850971	Chek1	checkpoint kinase 1	-2.3	6.11E-05
17795322	Clec2d2	C-type lectin domain family 2 member D2	-2.08	0.0006
17795347	Clec2h	C-type lectin domain family 2, member H	2.2	2.52E-06
17787572	Clec9a	C-type lectin domain family 9, member A	-2.01	1.19E-05
17799607	Cntln	centlein, centrosomal protein	-2.01	0.0009
17679536	Cntnap5c	contactin associated protein-like 5C	-3.2	0.0018
17754308	Col18a1	collagen, type XVIII, alpha 1	2	0.0024
17723923	Cplx4	complexin 4	5.29	0.0364
17666037	Csta	cystatin A (stefin A)	2.58	5.94E-08
17639564	Ctsw	cathepsin W	-2.12	8.25E-08
17800550	Cyb5rl	cytochrome b5 reductase-like	-2.07	4.54E-09
17614602	Cyp2a2	cytochrome P450, family 2, subfamily a, polypeptide 2	2.47	6.15E-08
17641925	Cyp2c24	cytochrome P450, family 2, subfamily c, polypeptide 24	-2.12	0.0045
17809417	Cyp4a2; LOC103690309	cytochrome P450, family 4, subfamily a, polypeptide 2; cytochrome P450 4A2-like; cytochrome P450, family 4, subfamily a, polypeptide 2 [Source:RGD Symbol;Acc:2479]	-2.41	0.0001
17809403	Cyp4a8	cytochrome P450, family 4, subfamily a, polypeptide 8	-2.68	6.81E-07
17848419	Dclk3	doublecortin-like kinase 3	-2.25	4.79E-05
17712670	Defb2	defensin beta 2	-2.16	4.78E-06
17712729	Defb14	defensin beta 14	2.04	3.27E-06
17692872	Dmp1	dentin matrix acidic phosphoprotein 1	2.38	1.22E-06
17821484	Dus4l	dihydrouridine synthase 4-like	-2.2	2.14E-07
17715279	Eci3	enoyl-Coenzyme A delta isomerase 3	-2.06	0.0008
17869380	Efcab1	EF hand calcium binding domain 1	-2.46	0.0003
17709486	Efnb2	ephrin B2	-2.15	0.0003
17872585	Fam47a	family with sequence similarity 47, member A	-2.02	1.54E-06
17719065	Fam50b	family with sequence similarity 50, member B	2.04	0.0004
17781980	Fam71f1	family with sequence similarity 71, member F1	-2.27	7.40E-09
17642433	Fgf8	fibroblast growth factor 8	-2.02	2.66E-06
17879250	Frmd7	FERM domain containing 7	-4.43	2.91E-05
17645401	Gfpt2	glutamine-fructose-6-phosphate transaminase 2	-2.08	0.0002

17783411	Gimap4	GTPase, IMAP family member 4	2.53	4.51E-07
17827288	Glt8d2	glycosyltransferase 8 domain containing 2	-2.12	0.0002
17705426	Gpr18	G protein-coupled receptor 18	-2.06	3.27E-05
17751479	Gstcd	glutathione S-transferase, C-terminal domain containing	-2	3.39E-06
17806402	Gzmb	granzyme B (granzyme 2, cytotoxic T-lymphocyte-associated serine esterase 1)	-2.15	2.96E-05
17749827	Hao2	hydroxyacid oxidase 2 (long chain)	-2.43	7.31E-06
17655129	Hba1	hemoglobin, alpha 1	5.77	1.00E-04
17655118	Hba-a1	hemoglobin alpha, adult chain 1	3.9	0.0015
17635606	Hbb	hemoglobin, beta	3.06	0.0058
17635628	Hbe2	hemoglobin, epsilon 2	2.55	1.42E-07
17635622	Hbg1	hemoglobin, gamma A	-2.35	0.0005
17788960	Hgf	hepatocyte growth factor	-2.2	0.0013
17750737	Hmox2-ps1	heme oxygenase (decycling) 2, pseudogene 1	-2.66	7.06E-09
17791409	Hoxa13	homeo box A13	2.51	6.76E-09
17781874	Hyal6	hyaluronoglucosaminidase 6	-2.09	1.45E-07
17876097	Hypm	huntingtin interacting protein M	-2.03	9.64E-08
17625123	Ifit1	interferon-induced protein with tetratricopeptide repeats 1	2.06	0.0002
17638626	Igf2	insulin-like growth factor 2	-2.5	0.0053
17613051	Il11	interleukin 11	-2.07	9.43E-06
17673846	Il31	interleukin 31	2.04	5.36E-05
17700517	Irg1	immunoresponsive gene 1	-2.45	5.30E-05
17632327	Klk1c6	kallikrein 1-related peptidase C6	3.11	2.02E-07
17795691	Klra1	killer cell lectin-like receptor, subfamily A, member 1	-2.14	0.0004
17787545	Klrb1	killer cell lectin-like receptor subfamily B, member 1	-2.35	1.45E-05
17795437	Klrc2; Klrc3	killer cell lectin-like receptor subfamily C, member 2; killer cell lectin-like receptor subfamily C, member 3	2.33	0.0006
17661169	Krtap16-1	keratin associated protein 16-1	-2.03	7.41E-08
17651028	Krtap31-1	keratin associated protein 31-1	2.17	1.54E-07
17749195	Lce1f	late cornified envelope 1F	-2.02	0.0305
17760207	Lcn1	lipocalin 1	-2.19	0.0051
17781961	Lep	leptin	-2.12	0.0001
17808217	LOC298139	similar to RIKEN cDNA 2310003M01	-2.14	3.41E-08
17869395	LOC363301	hypothetical LOC363301	2.64	1.50E-05
17653551	LOC363527	similar to serine/threonine protein kinase 6	-2.41	0.0005
17873455	LOC498424	similar to GC-rich promoter binding protein 1-like 1	-2.82	6.65E-09
17738224	LOC499607	similar to GTPase activating protein testicular GAP1	2.6	6.48E-06
17810051	LOC679803	similar to olfactory receptor Olr869	-2.76	5.50E-09
17873383	LOC680227	LRRGT00193	2.27	3.66E-08
17651020	LOC680428	hypothetical protein LOC680428	-2.63	0.0016
17694445	LOC680579	similar to ribosomal protein L14 [Source:RGD Symbol;Acc:1590581]	-2.24	0.0023
17728554	LOC685411	similar to spermatogenesis associated glutamate (E)-rich protein 4b [Source:RGD Symbol;Acc:1593845]	2.85	0.0034
17876865	LOC685699	hypothetical protein LOC685699	-2.1	5.10E-05
17693281	LOC685782	similar to PRAME family member 9	2.03	1.46E-05
17711886	LOC685829; Calcoco2	similar to calcium binding and coiled-coil domain 2; calcium binding and coiled-coil domain 2 [Source:MGI Symbol;Acc:MGI:1343177]	-2.49	0.0049
17774280	LOC686900; LOC103691843	similar to olfactory receptor 1198; olfactory receptor 4P4-like; similar to olfactory receptor 1198 [Source:RGD Symbol;Acc:1591313]; olfactory receptor 4P4-like [Source:RGD Symbol;Acc:9265291]	2.03	0.0011

17635600	LOC689064; LOC103694857; Hbb-b1	beta-globin; hemoglobin subunit beta-2; hemoglobin, beta adult major chain [Source:RGD Symbol;Acc:1595848]	2.68	0.0184
17809329	LOC689589	hypothetical protein LOC689589	-2.18	2.48E-07
17698203	LOC690384	similar to ribosomal protein L31 [Source:RGD Symbol;Acc:1597308]	-2.37	1.80E-07
17738904	LOC691044	similar to GTPase activating protein testicular GAP1	2.19	0.0009
17704136	LOC691988; LOC102550654	similar to a disintegrin and metalloproteinase domain 28; disintegrin and metalloproteinase domain-containing protein 28-like	2.41	6.33E-06
17681237	LOC100302372	hypothetical protein LOC100302372	-2.11	2.65E-08
17708505	LOC100359503	ribosomal protein S28-like	-2.08	4.49E-05
17792908	LOC100360057	ribosomal protein L22-like	-4.4	6.19E-07
17856633	LOC100360613	rCG25329-like	2.19	1.42E-08
17690708	LOC100362142	mCG20085-like	-3.22	2.79E-08
17832850	LOC100362350	hydroxysteroid 17-beta dehydrogenase 6-like; hydroxysteroid 17-beta dehydrogenase 6-like [Source:RGD Symbol;Acc:2318336]	2.12	7.64E-05
17854183	LOC100363141	dynein light chain 1-like	-2.29	0.0002
17696430	LOC100363290	hypothetical protein LOC100363290	-2.05	4.41E-08
17825058	LOC100363779	hypothetical protein LOC100363779 [Source:RGD Symbol;Acc:2322373]	-2.27	0.0106
17795777	LOC100364512	hypothetical LOC100364512	-2.63	2.18E-07
17807978	LOC100910017	60S ribosomal protein L31-like [Source:RGD Symbol;Acc:6500827]	-2.08	2.43E-05
17614267	LOC100910611	CD177 antigen-like; CD177 antigen-like [Source:RGD Symbol;Acc:6497684]	-2.32	0.0004
17857168	LOC100910668; LOC100911489	uncharacterized LOC100910668; uncharacterized LOC100911489	-2.27	3.58E-07
17737097	LOC100910852	uncharacterized LOC100910852	-2.8	2.23E-05
17666031	LOC100911790	stefin-3-like	3.08	2.50E-07
17824490	LOC100912557	6.8 kDa mitochondrial proteolipid-like	2.13	3.54E-05
17630502	LOC100912839	CD177 antigen-like	2.56	1.13E-07
17788338	LOC102547920	zinc finger protein 729-like	-2.55	0.0033
17860307	LOC102548134	uncharacterized LOC102548134	2.43	1.58E-09
17794653	LOC102550396	LRRGT00188	-2.6	4.47E-08
17613800	LOC102550682	carcinoembryonic antigen-related cell adhesion molecule 5-like	2.05	2.95E-08
17720404	LOC102550988; RGD1564999	isopentenyl-diphosphate delta-isomerase 2-like; similar to isopentenyl-diphosphate delta isomerase 2 [Source:RGD Symbol;Acc:1564999]	-3.08	6.38E-06
17872853	LOC102551431	profilin-2-like	2.03	1.27E-06
17697690	LOC102554774	disks large homolog 5-like	5.63	7.96E-07
17728146	LOC102557241	uncharacterized LOC102557241	-2.05	0.0019
17870238	LOC102557419; LOC301748; LOC685796; LOC363306; LOC685668; LOC100912904; LOC501224; LOC103690742	disks large homolog 5-like; similar to RIKEN cDNA 1700001E04; similar to similar to RIKEN cDNA 1700001E04; hypothetical protein LOC363306; hypothetical protein LOC685668; similar to RIKEN cDNA 2610042L04; uncharacterized LOC103690742	-2.09	0.0003
17615942	LOC103690048	prostatic glandular kallikrein-6-like	-2.3	0.0002
17633543	LOC103691126; LOC103691137	uncharacterized LOC103691126; uncharacterized LOC103691137	-2.32	0.0017
17620130	LOC103691230; LOC691519	putative ankyrin repeat domain-containing protein 19; similar to ankyrin repeat domain 26 [Source:RGD Symbol;Acc:1582795]	-2.18	1.95E-08
17794528	LOC103692218; LOC297568	murinoglobulin-1-like; alpha-1-inhibitor III [Source:RGD Symbol;Acc:1584999]	2.22	6.56E-05
17829032	LOC103692904	60S ribosomal protein L29-like	-2.52	3.43E-07
17701660	LOC103693372	histone H3.3-like	-2.72	2.65E-08

17659817	Lpo	lactoperoxidase	-2.01	3.32E-07
17667144	Lrrc74b	leucine rich repeat containing 74B	-2.38	0.0003
17835418	Lrriq1	leucine-rich repeats and IQ motif containing 1	-2.16	1.44E-05
17617281	Mcee	methylmalonyl CoA epimerase	-2.29	5.41E-08
17676422	MGC112692	similar to Leukosialin precursor (Leucocyte sialoglycoprotein) (Sialophorin) (CD43) (W3/13 antigen); similar to Leukosialin precursor (Leucocyte sialoglycoprotein) (Sialophorin) (CD43) (W3/13 antigen) [Source:RGD Symbol;Acc:1559964]	2.07	3.78E-05
17721894	MGC116121	similar to RIKEN cDNA 2700062C07	-2.08	5.14E-07
17714181	Mir7a-1	microRNA 7a-1	-2.1	0.0001
17858025	Mir30c2	microRNA 30c-2	-2.19	1.44E-05
17879338	Mir92a2	microRNA 92a-2	-2.31	1.36E-05
17856123	Mir128-2	microRNA 128-2	-2.29	1.86E-07
17774814	Mir129-2	microRNA 129-2	2.01	1.29E-06
17818435	Mir370	microRNA 370	-2.73	1.82E-05
17873013	Mir676	microRNA 676	-2.13	0.0004
17806396	Mir873	microRNA 873	-2.46	5.73E-05
17722380	Mir1949	microRNA 1949	2.1	0.0305
17799408	Mir3084c	microRNA 3084c	-2.19	4.66E-06
17834702	Mir3561	microRNA 3561	2.01	0.0017
17879671	Mir3585	microRNA 3585	-2.18	4.36E-09
17634145	Mir3597-3	microRNA 3597-3	2.61	5.70E-06
17714890	Mirlet7f-1	microRNA let-7f-1	-2.49	0.0049
17768956	Mmp9	matrix metallopeptidase 9	2.14	0.0025
17633297	Mrgprx2	MAS-related GPR, member X2	-2.02	4.37E-05
17821057	Msgn1	mesogenin 1	-2.01	8.01E-06
17724220	Myo5b	myosin Vb	-3.23	0.001
17629650	Ncr1	natural cytotoxicity triggering receptor 1	2.01	5.98E-06
17688286	Ndufb4l1	NADH dehydrogenase (ubiquinone) 1 beta subcomplex, 4-like 1 [Source:RGD Symbol;Acc:1560413]	-2.31	7.87E-07
17878914	Nxf3	nuclear RNA export factor 3	-2.34	2.49E-07
17634770	Olr19	olfactory receptor 19	2.11	2.82E-08
17634772	Olr24	olfactory receptor 24	3.05	2.80E-09
17619032	Olr41	olfactory receptor 41	-2.06	0.0003
17619070	Olr74	olfactory receptor 74	2.51	3.21E-06
17619080	Olr81	olfactory receptor 81	2.25	0.0009
17619134	Olr137	olfactory receptor 137	-2.59	4.71E-05
17635649	Olr145	olfactory receptor 145	2.1	4.25E-07
17635721	Olr176	olfactory receptor 176	2.96	2.74E-06
17635729	Olr183	olfactory receptor 183	-2.13	0.0001
17619216	Olr200	olfactory receptor 200	-2.95	0.0305
17619333	Olr230	olfactory receptor 230	2.16	0.0005
17635936	Olr232	olfactory receptor 232	-2.59	4.22E-05
17619401	Olr240	olfactory receptor 240	-2.28	3.25E-05
17619415	Olr260	olfactory receptor 260	-2.21	0.0016
17619445	Olr282	olfactory receptor 282	2.16	7.36E-06
17622009	Olr313	olfactory receptor 313	-2.49	0.0113
17640791	Olr318	olfactory receptor 318	2.09	0.0063
17640809	Olr331	olfactory receptor 331	2.24	1.05E-07
17624303	Olr338	olfactory receptor 338	2.16	0.003

17624359	Olr374	olfactory receptor 374	-2.45	3.76E-07
17771740	Olr421	olfactory receptor 421	-2.48	0.0021
17763564	Olr443	olfactory receptor 443	-2.84	0.0001
17763603	Olr472	olfactory receptor 472	-2.37	0.0278
17774222	Olr576	olfactory receptor 576	2.19	6.21E-05
17763696	Olr597	olfactory receptor 597	-3.08	0.0117
17763704	Olr606	olfactory receptor 606	2.07	0.0025
17774240	Olr608	olfactory receptor 608	-2.62	0.0066
17774260	Olr625	olfactory receptor 625	2.08	0.0017
17774266	Olr631	olfactory receptor 631	-4.38	1.00E-04
17763720	Olr635	olfactory receptor 635	-2.25	0.0004
17763742	Olr660	olfactory receptor 660	-2.34	7.17E-05
17774287	Olr661	olfactory receptor 661	2.91	4.59E-07
17774313	Olr677	olfactory receptor 677	2.33	1.68E-05
17774375	Olr721	olfactory receptor 721	-2.1	0.0003
17764621	Olr757	olfactory receptor 757	-2.01	7.41E-05
17764640	Olr773	olfactory receptor 773	2.35	2.17E-05
17775327	Olr790	olfactory receptor 790	-2.46	2.38E-05
17783081	Olr812; Olr437	olfactory receptor 812; olfactory receptor 437	-2.17	1.71E-06
17786437	Olr823	olfactory receptor 823	-2.17	0.0001
17786445	Olr827	olfactory receptor 827	-2.28	0.0052
17810061	Olr868	olfactory receptor 868	2.16	9.86E-08
17833185	Olr922	olfactory receptor 922	-2.14	0.0003
17825903	Olr996	olfactory receptor 996	2.23	1.83E-05
17834087	Olr1079	olfactory receptor 1079	2.43	0.0012
17826694	Olr1084	olfactory receptor 1084	2.3	2.67E-05
17842216	Olr1128	olfactory receptor 1128	2.25	0.0108
17849998	Olr1132	olfactory receptor 1132	-2.15	2.73E-05
17850002	Olr1137	olfactory receptor 1137	-2.76	7.08E-08
17842243	Olr1159	olfactory receptor 1159	-2.86	1.34E-05
17843221	Olr1196	olfactory receptor 1196	-2.05	0.0005
17843227	Olr1199	olfactory receptor 1199	-2.03	0.0005
17843235	Olr1203	olfactory receptor 1203	-2.09	0.004
17843262	Olr1222; LOC103693050	olfactory receptor 1222; olfactory receptor 143-like	-2.17	1.69E-06
17843280	Olr1232	olfactory receptor 1232	-2.07	0.0046
17843306	Olr1245	olfactory receptor 1245	2.18	0.0062
17843320	Olr1251	olfactory receptor 1251	2.15	7.23E-06
17843359	Olr1264	olfactory receptor 1264	2.1	0.0016
17843361	Olr1265; LOC100910111	olfactory receptor 1265; olfactory receptor 147-like	2.92	4.33E-08
17851262	Olr1293	olfactory receptor 1293	-3.42	1.98E-06
17851315	Olr1304	olfactory receptor 1304	-2.28	0.0045
17843379	Olr1308	olfactory receptor 1308	-3.62	2.97E-09
17843409	Olr1329	olfactory receptor 1329	2.69	6.83E-09
17654044	Olr1362	olfactory receptor 1362	-2.13	0.0089
17646233	Olr1456	olfactory receptor 1456	2.25	7.45E-05
17646235	Olr1458	olfactory receptor 1458	-2.38	0.0007
17646237	Olr1459	olfactory receptor 1459	-2.25	0.0028
17658502	Olr1500	olfactory receptor 1500	2.37	0.0004

17648368	Olr1511	olfactory receptor 1511	-2.56	0.0002
17648374	Olr1515	olfactory receptor 1515	-2.27	1.51E-05
17664200	Olr1525; Olr1355	olfactory receptor 1525; olfactory receptor 1355	2.03	5.44E-08
17665114	Olr1532	olfactory receptor 1532	-2.06	0.0151
17665118	Olr1539	olfactory receptor 1539	2.2	0.0077
17665138	Olr1551	olfactory receptor 1551	4.39	2.38E-06
17698058	Olr1611	olfactory receptor 1611	2.81	2.92E-05
17702226	Olr1645	olfactory receptor 1645	-2.54	0.0006
17713690	Olr1652	olfactory receptor 1652	-2.84	1.26E-07
17732758	Olr1667	olfactory receptor 1667	-2.12	4.71E-07
17755924	Olr1686; LOC100910479	olfactory receptor gene Olr1686; olfactory receptor 2G3-like; olfactory receptor 2G3-like [Source:RGD Symbol;Acc:6498308]	2.17	5.63E-05
17752327	Olr1748	olfactory receptor 1748	-2.11	1.01E-07
17840677	Or10ad1	olfactory receptor, family 10, subfamily AD, member 1	-2.14	3.05E-07
17739188	Otol1	otolin 1	2.3	2.73E-05
17873718	Pcdh11x	protocadherin 11 X-linked; protocadherin 11 X-linked [Source:RGD Symbol;Acc:1562864]	-2.55	4.25E-05
17733853	Pkd1l2	polycystic kidney disease 1-like 2	2.12	8.21E-07
17725948	Plac8l1	PLAC8-like 1	-2.09	5.49E-06
17610530	Plagl1	pleiomorphic adenoma gene-like 1	-2.23	4.10E-06
17611414	Ppp1r14c	protein phosphatase 1, regulatory (inhibitor) subunit 14c	-2.07	1.15E-05
17652944	Prcd	progressive rod-cone degeneration	-2.23	3.55E-06
17719265	Prl3d4	prolactin family 3, subfamily d, member 4	-2.91	2.80E-08
17719226	Prl5a2	prolactin family 5, subfamily a, member 2	2	1.48E-06
17782888	Prss2	protease, serine, 2	2.16	7.35E-05
17721635	Psma8	proteasome subunit alpha 8	-2.11	0.0002
17636351	Pth	parathyroid hormone	-2.01	1.30E-08
17680319	Rabif; RGD1563962	RAB interacting factor; similar to Mss4 protein	-2.03	5.44E-06
17789835	Rbmxl1	RNA binding motif protein, X-linked-like 1	-2.27	1.29E-05
17850921	RGD1304554	LOC363043	2.25	8.40E-09
17835987	RGD1306474	similar to RIKEN cDNA 9530003J23	-2.2	0.0002
17645700	RGD1306484	similar to growth and transformation-dependent protein [Source:RGD Symbol;Acc:1306484]	2.1	0.0007
17861243	RGD1311447	LOC363276	-2.03	1.41E-05
17641180	RGD1359158	similar to RIKEN cDNA 1110059E24	3	0.0092
17822953	RGD1559921	similar to Macrophage migration inhibitory factor (MIF) (Delayed early response protein 6) [Source:RGD Symbol;Acc:1559921]	-2.21	5.71E-08
17856901	RGD1559960	similar to Sulfotransferase K1 (rSULT1C2)	-2.11	0.0328
17827340	RGD1560034	similar to FLJ25323 protein	-2.21	1.18E-08
17850967	RGD1560112	similar to Urinary protein 2 precursor (RUP-2)	2.26	3.70E-08
17759240	RGD1561147	similar to hypothetical protein FLJ37396	-2.1	5.74E-06
17808105	RGD1561195	similar to ribosomal protein L31 [Source:RGD Symbol;Acc:1561195]	-3.03	2.04E-08
17726231	RGD1561627	similar to hypothetical protein 4930474N05	-2.13	0.0008
17747217	RGD1561841	similar to ribosomal protein L31 [Source:RGD Symbol;Acc:1561841]	-2.22	0.0014
17752465	RGD1562652	similar to class I histocompatibility antigen alpha chain - cotton-top tamarin	2.29	1.08E-08
17690129	RGD1562755	similar to 60S ribosomal protein L23a [Source:RGD Symbol;Acc:1562755]	-2.28	0.0063
17631873	RGD1563307	similar to Set beta isoform	-2.07	8.93E-06
17876019	RGD1564613	similar to MGC40405 protein	-2.14	0.0129

17821087	RGD1565679	similar to 60S ribosomal protein L27a (L29)	-2.01	0.0003
17870530	Rhox9	reproductive homeobox 9	2.09	4.53E-06
17611232	Rspo3	R-spondin 3	-2.26	0.0051
17699332	Sap18	Sin3-associated polypeptide 18	-2.06	0.014
17642329	Scd1	stearoyl-Coenzyme A desaturase 1	-2.09	0.0225
17623944	Scgb2a2	secretoglobin, family 2A, member 2	-2.14	0.0003
17715387	Serpinb1a	serine (or cysteine) proteinase inhibitor, clade B, member 1a	-3.45	1.74E-06
17774023	Serping1	serpin peptidase inhibitor, clade G (C1 inhibitor), member 1	2.16	0.0236
17880129	Sh3bp4	SH3-domain binding protein 4	-2.01	0.0001
17722829	Sh3rf2	SH3 domain containing ring finger 2	-2.13	3.73E-06
17657978	Slc2a4	solute carrier family 2 (facilitated glucose transporter), member 4	-2.04	4.16E-09
17786689	Slc6a13	solute carrier family 6 (neurotransmitter transporter), member 13	-2.47	7.25E-08
17741804	Slc16a4	solute carrier family 16, member 4	-2.03	2.69E-07
17853485	Slc24a1	solute carrier family 24 (sodium/potassium/calcium exchanger), member 1	-2.01	1.87E-06
17844204	Sln	sarcolipin	2.02	0.0015
17626378	Sorcs3	sortilin-related VPS10 domain containing receptor 3	2.02	0.0323
17657890	Spem1	spermatid maturation 1	-2.32	9.89E-07
17724130	Stard6	StAR-related lipid transfer (START) domain containing 6	-2.18	3.72E-07
17825331	Sult6b1	sulfotransferase family 6B member 1	-2.06	0.0007
17807697	Susd1	sushi domain containing 1	-2.35	0.0005
17779169	Svs3b	seminal vesicle secretory protein 3B	2.18	0.0008
17827431	Sycp3	synaptonemal complex protein 3	-2.36	1.59E-05
17611054	Taar8b	trace amine-associated receptor 8b	-3.68	7.18E-06
17628910	Tcte3	t-complex-associated testis expressed 3	2.01	6.12E-08
17653382	Tex19.1	testis expressed 19.1	-2.36	0.0001
17685675	Tex35	testis expressed 35	-2.12	1.20E-06
17754639	Tfam	transcription factor A, mitochondrial	-2.03	1.24E-06
17776215	Tgm7l1	transglutaminase 7-like 1	-2.04	3.60E-07
17629582	Tmem190	transmembrane protein 190	-2.11	3.63E-07
17700148	Tpt1	tumor protein, translationally-controlled 1	-6.46	0.0021
17829224	Trhr	thyrotropin releasing hormone receptor	-2.55	0.009
17734101	Trhr2	thyrotropin releasing hormone receptor 2	-2.15	3.67E-06
17700596	Trim52	tripartite motif-containing 52	2.01	3.33E-07
17795006	Tuba3a	tubulin, alpha 3A	-2.59	1.29E-07
17640224	Ust5r	integral membrane transport protein UST5r	2.38	2.26E-05
17629508	Vom1r35	vomeronasal 1 receptor 35	-2.07	0.0001
17613318	Vom1r44	vomeronasal 1 receptor 44	-2.29	1.93E-07
17783877	Vom1r65	vomeronasal 1 receptor 65	-2.41	5.52E-07
17783883	Vom1r68	vomeronasal 1 receptor 68	-2.38	1.86E-05
17783891	Vom1r72	vomeronasal 1 receptor 72	2.12	3.21E-08
17826910	Vom1r107	vomeronasal 1 receptor 107	2.11	8.27E-07
17612614	Vom2r21	vomeronasal 2 receptor, 21	2.26	2.31E-07
17692389	Vom2r73; Vom2r-ps125	vomeronasal 2 receptor, 73; vomeronasal 2 receptor, pseudogene 125	-2.18	9.12E-07
17860210	Vwc2l	von Willebrand factor C domain-containing protein 2-like	-2.1	4.58E-09
17751879	Wdr63	WD repeat domain 63	-2.09	2.34E-06
17779226	Wfdc6a	WAP four-disulfide core domain 6A	-2.39	2.11E-07
17670864	Ypel1	yippee-like 1	-2.18	2.82E-05
17612499	Zfp53	zinc finger protein 53	-2.99	3.08E-06

17612922	Zim1	zinc finger, imprinted 1	-2.25	7.18E-07
17870180		Sequence without official gene name	-2.48	4.85E-08
17881725		Sequence without official gene name	-2.68	0.0001
17820577		Sequence without official gene name	-2.83	0.003
17881751		Sequence without official gene name	-2.65	0.0001
17881755		Sequence without official gene name	-2.45	0.0001
17880825		Sequence without official gene name	-2.62	0.0058
17881935		Sequence without official gene name	2.28	1.37E-07
17869474		Sequence without official gene name	2	0.0122
17881949		Sequence without official gene name	2.15	2.26E-08
17820391		Sequence without official gene name	-2.25	4.84E-09
17818965		Sequence without official gene name	-2.76	0.0128
17860219		Sequence without official gene name	-2.28	3.19E-05
17869483		Sequence without official gene name	-2.57	8.05E-05
17862387		Sequence without official gene name	-2.08	0.0017
17822145		Sequence without official gene name	2.25	1.37E-05
17868400		Sequence without official gene name	-2.1	0.0003
17821588		Sequence without official gene name	-2.11	2.16E-06
17882255		Sequence without official gene name	-2.61	3.42E-09
17881535		Sequence without official gene name	-2.68	0.0001
17881503		Sequence without official gene name	-2.09	0.0233
17870231		Sequence without official gene name	-2.85	1.84E-08
17869980		Sequence without official gene name	-2.18	5.69E-05
17880991		Sequence without official gene name	2.27	0.0228
17880931		Sequence without official gene name	-2.31	4.37E-09
17870157		Sequence without official gene name	-2.26	8.63E-05
17821349		Sequence without official gene name	-2.32	6.15E-10
17821089		Sequence without official gene name	-2.36	0.0024
17838118		Sequence without official gene name	-2.49	6.10E-06
17817128		Sequence without official gene name	-3.73	6.83E-09
17882483		Sequence without official gene name	-2.1	2.02E-07
17883105		Sequence without official gene name	-3.09	8.89E-06
17883143		Sequence without official gene name	-2.13	0.0015
17883187		Sequence without official gene name	-2.46	0.0017
17810041		Sequence without official gene name	-2.6	0.003
17809820		Sequence without official gene name	-2.18	3.16E-05
17851973		Sequence without official gene name	-2.97	2.97E-05
17810158		Sequence without official gene name	2.98	5.39E-09
17852458		Sequence without official gene name	-2.33	6.27E-06
17883193		Sequence without official gene name	-2.06	0.0008
17858097		Sequence without official gene name	4.79	3.24E-07
17808756		Sequence without official gene name	-2.14	9.80E-05
17869467		Sequence without official gene name	-2.26	8.63E-05
17883197		Sequence without official gene name	-4.1	0.0131
17853483		Sequence without official gene name	2.72	5.30E-05
17869469		Sequence without official gene name	2.11	0.0093
17859208		Sequence without official gene name	2.37	4.82E-05
17810662		Sequence without official gene name	-2.69	4.02E-05
17815154		Sequence without official gene name	-2.49	8.25E-06
17818428		Sequence without official gene name	-2.44	0.0029

17880818	Sequence without official gene name	-2.45	1.33E-07
17817028	Sequence without official gene name	2.42	6.31E-08
17816070	Sequence without official gene name	-2.06	2.82E-05
17882519	Sequence without official gene name	-2.31	7.14E-07
17815960	Sequence without official gene name	-2.35	8.12E-05
17810710	Sequence without official gene name	2.22	0.0005
17882603	Sequence without official gene name	-2.08	0.0019
17882655	Sequence without official gene name	-2.42	0.009
17868698	Sequence without official gene name	-2.68	0.0005
17882723	Sequence without official gene name	-2.04	8.15E-06
17882789	Sequence without official gene name	2.44	0.0009
17882845	Sequence without official gene name	2.17	0.0002
17882903	Sequence without official gene name	2.75	3.11E-06
17815528	Sequence without official gene name	-2.01	0.0143
17880794	Sequence without official gene name	-2.36	0.0008
17880650	Sequence without official gene name	-2.86	6.33E-07
17880683	Sequence without official gene name	-2.21	8.93E-06
17870411	Sequence without official gene name	-2.06	0.0043
17829201	Sequence without official gene name	2.35	0.0075
17828431	Sequence without official gene name	-2.06	0.0017
17828030	Sequence without official gene name	-2.82	3.35E-07
17870356	Sequence without official gene name	2	3.95E-06
17844933	Sequence without official gene name	-2.31	0.0014
17867322	Sequence without official gene name	-2.2	0.0211
17876456	Sequence without official gene name	-2.62	5.35E-07
17827056	Sequence without official gene name	-3	6.91E-06
17876491	Sequence without official gene name	-2.53	0.0022
17877418	Sequence without official gene name	-2.08	3.88E-06
17877689	Sequence without official gene name	-2.06	0.0008
17877691	Sequence without official gene name	2.3	1.74E-07
17825947	Sequence without official gene name	-2.34	4.33E-06
17878279	Sequence without official gene name	-2.02	2.03E-05
17874787	Sequence without official gene name	-2.11	4.13E-05
17825905	Sequence without official gene name	-2.13	5.17E-05
17874408	Sequence without official gene name	-2.23	0.0008
17873741	Sequence without official gene name	2.17	0.0016
17837611	Sequence without official gene name	-2.13	0.0016
17836781	Sequence without official gene name	-2.75	3.41E-07
17836377	Sequence without official gene name	-2.02	0.0023
17867518	Sequence without official gene name	-2.23	2.28E-06
17867336	Sequence without official gene name	-3.4	0.0177
17871315	Sequence without official gene name	-2.16	1.20E-05
17871554	Sequence without official gene name	-2.01	0.0002
17873597	Sequence without official gene name	-2.54	3.42E-05
17843984	Sequence without official gene name	2.34	0.0351
17873683	Sequence without official gene name	-2.54	7.65E-05
17833220	Sequence without official gene name	2.31	0.0002
17867332	Sequence without official gene name	-2.74	0.0001
17833208	Sequence without official gene name	-2.26	3.99E-06
17867330	Sequence without official gene name	-2.71	0.0132

17833177	Sequence without official gene name	-2.05	1.25E-05
17830322	Sequence without official gene name	-2.68	1.02E-05
17880756	Sequence without official gene name	-2.1	0.0027
17878839	Sequence without official gene name	-2.18	5.61E-05
17825068	Sequence without official gene name	-2.09	1.28E-05
17880438	Sequence without official gene name	2.51	4.13E-07
17865227	Sequence without official gene name	-3.28	0.0005
17823480	Sequence without official gene name	-2.59	9.20E-05
17823474	Sequence without official gene name	-2.18	1.55E-05
17868798	Sequence without official gene name	2.03	0.0043
17823051	Sequence without official gene name	2.66	2.95E-06
17880454	Sequence without official gene name	-2.18	0.0084
17863211	Sequence without official gene name	2.02	2.18E-07
17822828	Sequence without official gene name	-2.06	4.88E-07
17822216	Sequence without official gene name	-2.24	2.16E-06
17862605	Sequence without official gene name	-2.46	1.79E-06
17880463	Sequence without official gene name	-2.03	5.37E-08
17880517	Sequence without official gene name	-2	1.57E-07
17862389	Sequence without official gene name	-2.74	0.0359
17840408	Sequence without official gene name	-2.68	1.48E-05
17849723	Sequence without official gene name	-2.11	0.0264
17840437	Sequence without official gene name	2.02	3.54E-06
17880428	Sequence without official gene name	-2.23	5.58E-05
17880416	Sequence without official gene name	-2.24	4.43E-09
17808685	Sequence without official gene name	-2.17	1.24E-05
17879065	Sequence without official gene name	2.09	0.0332
17879497	Sequence without official gene name	2.85	2.34E-07
17868261	Sequence without official gene name	-2.03	0.0034
17846276	Sequence without official gene name	-2.03	0.0001
17824848	Sequence without official gene name	-2.91	6.89E-08
17842170	Sequence without official gene name	-3.4	1.47E-09
17824820	Sequence without official gene name	-2.47	1.10E-05
17824773	Sequence without official gene name	-2.62	2.61E-07
17824656	Sequence without official gene name	-2.9	6.41E-07
17866865	Sequence without official gene name	-2.19	3.92E-08
17879589	Sequence without official gene name	-2.37	5.06E-07
17866177	Sequence without official gene name	-2.01	6.22E-06
17870260	Sequence without official gene name	2.05	0.0002
17849245	Sequence without official gene name	-3.3	2.87E-10
17849660	Sequence without official gene name	2.07	0.0002
17856338	Sequence without official gene name	-2.61	3.75E-08
17610302	Sequence without official gene name	2.01	0.0018
17808186	Sequence without official gene name	-2.06	5.12E-07
17686113	Sequence without official gene name	2.53	0.009
17684940	Sequence without official gene name	3.02	5.98E-08
17683534	Sequence without official gene name	2.23	2.47E-06
17683505	Sequence without official gene name	2.72	0.0021
17683384	Sequence without official gene name	-2.33	2.19E-07
17682315	Sequence without official gene name	-2.31	0.0363
17681711	Sequence without official gene name	-2.09	0.0003

17681110	Sequence without official gene name	2.1	8.55E-05
17681102	Sequence without official gene name	2.46	0.0011
17680711	Sequence without official gene name	2.02	1.77E-05
17679869	Sequence without official gene name	-2.15	2.72E-05
17689282	Sequence without official gene name	-3.25	1.56E-06
17679671	Sequence without official gene name	-2.78	7.92E-05
17679284	Sequence without official gene name	-2.5	4.68E-06
17679233	Sequence without official gene name	3.29	4.85E-07
17676941	Sequence without official gene name	2.13	1.67E-07
17675009	Sequence without official gene name	-2.05	7.55E-07
17674327	Sequence without official gene name	2.05	0.0005
17674147	Sequence without official gene name	2.18	0.0005
17672484	Sequence without official gene name	2.77	9.73E-08
17670118	Sequence without official gene name	-2.15	0.0001
17670112	Sequence without official gene name	-2.37	5.86E-05
17670072	Sequence without official gene name	-2.07	0.0078
17669851	Sequence without official gene name	2.16	4.23E-05
17679501	Sequence without official gene name	-2.66	3.23E-05
17669427	Sequence without official gene name	-2.18	5.33E-05
17689721	Sequence without official gene name	2.37	0.0001
17690433	Sequence without official gene name	-2.16	1.01E-06
17702336	Sequence without official gene name	-2.15	1.40E-05
17702328	Sequence without official gene name	2.29	3.44E-07
17702293	Sequence without official gene name	-2.09	5.21E-07
17702284	Sequence without official gene name	-4.12	1.38E-09
17702085	Sequence without official gene name	-2.27	1.16E-07
17701909	Sequence without official gene name	-2.11	7.24E-05
17701560	Sequence without official gene name	-2.09	0.0002
17701193	Sequence without official gene name	-2.41	4.76E-06
17700898	Sequence without official gene name	2.06	0.0002
17700834	Sequence without official gene name	2.08	3.05E-08
17699647	Sequence without official gene name	-2.32	2.63E-06
17689892	Sequence without official gene name	-2.32	3.31E-08
17699104	Sequence without official gene name	2.32	0.0347
17698427	Sequence without official gene name	-2.31	4.12E-07
17697608	Sequence without official gene name	-2.04	5.25E-07
17697343	Sequence without official gene name	2.43	2.17E-06
17696972	Sequence without official gene name	2.35	9.39E-06
17696724	Sequence without official gene name	-2.31	2.88E-05
17696482	Sequence without official gene name	2.53	0.0002
17695867	Sequence without official gene name	-2.23	0.0386
17694807	Sequence without official gene name	-2.21	0.0009
17694441	Sequence without official gene name	-2.11	0.0021
17693882	Sequence without official gene name	-2.01	4.68E-07
17692372	Sequence without official gene name	2.43	0.0005
17698486	Sequence without official gene name	2.5	7.07E-07
17703273	Sequence without official gene name	-2.13	0.0002
17669398	Sequence without official gene name	-2.63	4.96E-09
17668155	Sequence without official gene name	2.03	5.48E-05
17631902	Sequence without official gene name	-2.14	3.56E-05

17628759	Sequence without official gene name	2.4	3.22E-07
17628729	Sequence without official gene name	-2.33	2.78E-07
17628509	Sequence without official gene name	2.03	0.0023
17628461	Sequence without official gene name	-2.26	0.0001
17627560	Sequence without official gene name	2.11	4.89E-09
17627073	Sequence without official gene name	-2.27	0.0053
17626509	Sequence without official gene name	-2.18	3.19E-06
17626408	Sequence without official gene name	2.64	6.30E-07
17625166	Sequence without official gene name	2.55	2.75E-08
17624370	Sequence without official gene name	-2.16	0.0014
17633551	Sequence without official gene name	-2.59	1.38E-06
17623681	Sequence without official gene name	-2.56	2.61E-06
17621985	Sequence without official gene name	-2.02	0.0007
17619536	Sequence without official gene name	-2.42	0.0057
17619452	Sequence without official gene name	-2.48	0.0001
17617825	Sequence without official gene name	-2.08	0.0457
17617129	Sequence without official gene name	-2.01	1.70E-05
17616903	Sequence without official gene name	-2.5	1.32E-06
17612580	Sequence without official gene name	2.09	0.0352
17612377	Sequence without official gene name	-2.99	0.0009
17611551	Sequence without official gene name	-2.11	0.0264
17610773	Sequence without official gene name	2.67	0.0451
17610381	Sequence without official gene name	2.17	0.0002
17623677	Sequence without official gene name	-2	0.0034
17669060	Sequence without official gene name	-2.2	0.0053
17633611	Sequence without official gene name	-2.11	0.0023
17635561	Sequence without official gene name	-4.23	4.71E-07
17667289	Sequence without official gene name	-2.26	0.0001
17666202	Sequence without official gene name	-2.03	0.0012
17666024	Sequence without official gene name	-2.96	2.34E-10
17666006	Sequence without official gene name	2.15	2.96E-07
17665501	Sequence without official gene name	2.02	0.0007
17665312	Sequence without official gene name	-2.64	2.03E-06
17665061	Sequence without official gene name	-2.54	3.09E-06
17664495	Sequence without official gene name	-2.35	0.0006
17664320	Sequence without official gene name	-2.43	7.79E-08
17664249	Sequence without official gene name	2.8	4.20E-06
17662642	Sequence without official gene name	-2.17	0.0003
17633645	Sequence without official gene name	4.27	1.23E-07
17661045	Sequence without official gene name	-2.15	1.57E-08
17660034	Sequence without official gene name	-2.21	3.95E-08
17655720	Sequence without official gene name	-2.29	3.03E-06
17655103	Sequence without official gene name	-2.02	0.0004
17654238	Sequence without official gene name	-2.46	1.83E-07
17647241	Sequence without official gene name	-2.16	0.0059
17643356	Sequence without official gene name	-2.54	1.99E-05
17641419	Sequence without official gene name	2.22	0.0248
17638525	Sequence without official gene name	2.09	0.0052
17637184	Sequence without official gene name	2.49	2.97E-08
17636532	Sequence without official gene name	-2.09	2.40E-06

17635984	Sequence without official gene name	-2.38	0.0024
17660673	Sequence without official gene name	-2.2	0.0003
17808229	Sequence without official gene name	2.09	0.0003
17703387	Sequence without official gene name	-2.59	2.59E-08
17707443	Sequence without official gene name	2.35	4.73E-05
17775623	Sequence without official gene name	-2.27	1.83E-06
17773315	Sequence without official gene name	-2.09	0.0048
17773289	Sequence without official gene name	-2.16	4.42E-08
17772363	Sequence without official gene name	-2.1	0.0001
17770715	Sequence without official gene name	2.2	5.11E-06
17766508	Sequence without official gene name	-2.52	4.75E-08
17766359	Sequence without official gene name	2.04	7.28E-07
17760328	Sequence without official gene name	-2.35	1.67E-06
17757581	Sequence without official gene name	-2.49	0.0372
17757392	Sequence without official gene name	-2.37	6.00E-06
17756113	Sequence without official gene name	-2.2	0.0002
17777749	Sequence without official gene name	2.14	0.008
17755248	Sequence without official gene name	2.09	0.003
17752439	Sequence without official gene name	-2.38	9.32E-08
17752423	Sequence without official gene name	-2.08	2.41E-05
17883199	Sequence without official gene name	2.76	0.0009
17751562	Sequence without official gene name	-2.65	4.87E-06
17751462	Sequence without official gene name	-2.5	0.0034
17751458	Sequence without official gene name	-2.49	1.58E-07
17751015	Sequence without official gene name	-2.02	0.0014
17748743	Sequence without official gene name	-2.41	2.90E-06
17747778	Sequence without official gene name	-3.41	8.40E-07
17747033	Sequence without official gene name	2.28	0.0014
17746564	Sequence without official gene name	-2.4	2.84E-09
17755171	Sequence without official gene name	-2.27	0.0173
17746419	Sequence without official gene name	-2.88	3.85E-05
17780855	Sequence without official gene name	2.1	6.60E-05
17784265	Sequence without official gene name	-2.41	0.0006
17806985	Sequence without official gene name	2.22	7.95E-06
17806771	Sequence without official gene name	2.06	0.0084
17805274	Sequence without official gene name	-2.4	3.81E-06
17804684	Sequence without official gene name	-2.1	0.0007
17804096	Sequence without official gene name	-2.95	2.60E-06
17802828	Sequence without official gene name	-2.08	1.30E-06
17799940	Sequence without official gene name	-2.08	0.0021
17799873	Sequence without official gene name	2.89	2.56E-08
17798353	Sequence without official gene name	-2.18	7.98E-05
17797625	Sequence without official gene name	2.01	0.0288
17797336	Sequence without official gene name	-2.01	0.002
17781049	Sequence without official gene name	-2.15	2.13E-05
17796574	Sequence without official gene name	-2.04	7.26E-05
17793415	Sequence without official gene name	-3.03	0.0009
17791996	Sequence without official gene name	-2.09	4.44E-09
17791971	Sequence without official gene name	2.14	0.0013
17791968	Sequence without official gene name	-2.11	0.0001

17791927	Sequence without official gene name	-2.88	1.21E-07
17791267	Sequence without official gene name	-2.41	0.0004
17788910	Sequence without official gene name	-2.1	7.71E-05
17788736	Sequence without official gene name	-2.4	0.0006
17785142	Sequence without official gene name	-2.07	2.01E-08
17784312	Sequence without official gene name	2.54	2.49E-06
17784303	Sequence without official gene name	-2.11	8.28E-08
17794650	Sequence without official gene name	2.27	5.84E-08
17703425	Sequence without official gene name	-3.05	0.0063
17746192	Sequence without official gene name	-2.45	1.65E-07
17745523	Sequence without official gene name	-2.12	3.21E-06
17724530	Sequence without official gene name	-2.05	1.09E-05
17724527	Sequence without official gene name	-2.31	4.13E-06
17723310	Sequence without official gene name	-2.36	1.15E-05
17722817	Sequence without official gene name	2.89	4.20E-06
17722256	Sequence without official gene name	-2.11	0.0017
17721995	Sequence without official gene name	2.27	1.02E-05
17721677	Sequence without official gene name	-2.23	0.0459
17720702	Sequence without official gene name	2.13	7.89E-05
17720670	Sequence without official gene name	-2.03	0.0365
17720028	Sequence without official gene name	-2.28	0.0009
17718769	Sequence without official gene name	-2.14	0.0002
17724901	Sequence without official gene name	-3.83	2.73E-07
17717859	Sequence without official gene name	-2.16	0.0011
17715648	Sequence without official gene name	-2.14	3.01E-05
17715057	Sequence without official gene name	-2.31	1.36E-07
17714318	Sequence without official gene name	-2.68	0.0011
17712592	Sequence without official gene name	-2.36	6.75E-08
17712422	Sequence without official gene name	-2	0.0004
17711394	Sequence without official gene name	2.17	1.53E-06
17711295	Sequence without official gene name	2.64	4.34E-07
17711235	Sequence without official gene name	-2.73	0.0069
17710348	Sequence without official gene name	-2.39	2.82E-06
17709864	Sequence without official gene name	-2.04	0.0009
17708503	Sequence without official gene name	-2.21	4.51E-06
17717621	Sequence without official gene name	-2.13	0.0014
17745668	Sequence without official gene name	-2.08	4.11E-05
17725568	Sequence without official gene name	-2.04	0.0091
17726392	Sequence without official gene name	2.05	1.66E-05
17745459	Sequence without official gene name	2.4	0.0003
17744454	Sequence without official gene name	-2.39	1.46E-06
17744006	Sequence without official gene name	-2.05	0.0041
17743913	Sequence without official gene name	-2.14	2.00E-06
17743572	Sequence without official gene name	-2.23	2.29E-07
17743570	Sequence without official gene name	-2.48	0.0003
17741514	Sequence without official gene name	2.89	0.0026
17740451	Sequence without official gene name	-2.06	5.60E-06
17738213	Sequence without official gene name	2.17	0.0118
17737314	Sequence without official gene name	-2.38	0.0052
17737220	Sequence without official gene name	2.09	0.0014

17725931	Sequence without official gene name	2.05	5.57E-05
17737014	Sequence without official gene name	-3.63	0.0001
17736646	Sequence without official gene name	-2.41	0.0384
17736023	Sequence without official gene name	2.1	0.0002
17736021	Sequence without official gene name	-2.37	1.78E-05
17732712	Sequence without official gene name	-2.08	1.65E-05
17731388	Sequence without official gene name	-2.15	1.48E-05
17731112	Sequence without official gene name	-2.18	3.12E-06
17730502	Sequence without official gene name	-2.11	8.93E-06
17730400	Sequence without official gene name	-2.03	1.10E-06
17728515	Sequence without official gene name	-2.15	0.0021
17728362	Sequence without official gene name	-2.22	0.0013
17727514	Sequence without official gene name	-2.1	7.90E-05
17736687	Sequence without official gene name	-2.48	0.0001
17883207	Sequence without official gene name	-2.46	0.0006
